# A Bioenergetic Framework for Microplastic Accumulation in Human Tissues: A Cellular Turnover Hypothesis

**DOI:** 10.3390/toxics14070603

**Published:** 2026-07-10

**Authors:** Umberto Cornelli, Giuseppe Zanoni, Claudio Casella

**Affiliations:** 1 Department of Molecular Pharmacology and Therapeutics, School of Medicine, Loyola University, 2160 1st Ave, Maywood, IL 60660, USA; 2 Department of Chemistry, University of Pavia, Viale Taramelli 12, 27100 Pavia, Italy; gz@unipv.it (G.Z.); claudio.casella01@universitadipavia.it (C.C.)

**Keywords:** micro-nanoplastics, bioenergetic vulnerability, microplastic-induced syndrome (MI-Syn), Chicago cluster, lactate dehydrogenase, C-reactive protein

## Abstract

Micro- and nanoplastics (MNPs) are now pervasive in human tissues, yet their biological behavior remains unexplained within conventional pharmacokinetic frameworks. Here, we propose that MNP distribution may follow a bioenergetic logic governed by cellular turnover and metabolic demand, rather than passive diffusion alone. Integrating the human autopsy literature datasets with programmatic biological parameters suggests that MNPs persist intracellularly and are propagated through cycles of cell death and renewal, establishing a previously unrecognized system of retention-driven recirculation. By integrating tissue-specific metabolic rates, macrophage abundance, and intracellular vulnerability indices across 19 organs, we define a hierarchy of susceptibility, with highest accumulation in the spleen, intestinal epithelium, lung, and bone marrow. This hierarchy maps onto clinical patterns of tissue dysfunction and supports a unifying mechanism in which oxidative stress, energetic instability, and chronic inflammation emerge as convergent responses to MNP burden. We further identify a minimal circulating signature—lactate, high-sensitivity C-reactive protein (hsCRP), and lactate dehydrogenase (LDH)—that reflects systemic bioenergetic disruption associated with MNP exposure. Together, this framework offers a conceptual shift from diffusion-limited to turnover-driven accumulation models, providing testable hypotheses for future prospective validation.

## 1. Introduction

Synthetic materials are becoming more and more incorporated into the planet’s biology as humanity enters a new era. Every year, more than 450 million tons of plastic are generated [1], roughly 54 kg per person per year, providing an ongoing and inevitable burden of micro- and nanoplastics (MNPs). Consequently, human populations are chronically exposed to particulate polymers at a rate and scale that fundamentally outpaces homeostatic biological adaptation. In contrast to naturally existing biological macromolecules, MNPs currently exhibit extended biological persistence once internalized and lack recognized human enzymatic breakdown mechanisms [2,3,4]. MNPs have been identified in human blood [5,6,7] and in several types of substantial tissues, such as the kidney, liver, lung, myocardium, and thrombi [8,9,10]. A common pattern appears in all species, including humans, rats, and livestock: blood concentrations are usually in the ng/mL range, whereas tissue concentrations are 10–1000 times greater, reaching ng/g to µg/g concentrations (Table 1).

Classical pharmacokinetics parameters fail to accommodate this unique tissue distribution. While larger MPs are structurally constrained by anatomical barriers, an increasing body of evidence demonstrates that NPs actively cross complex biological membranes. This unique tissue distribution cannot be accommodated by traditional pharmacokinetic parameters. A growing amount of data demonstrates that NPs actively traverse complicated biological membranes, whereas bigger MPs are physically limited by anatomical obstacles. Systemic access to privileged cellular compartments is made possible by this translocation, which takes place via paracellular transport via broken tight junctions, endocytosis driven by clathrin or caveolae, and passive lipid bilayer rupture. Long-term lysosomal entrapment and minimally active exocytosis cause MNPs to stay trapped inside cells until apoptosis induces interstitial release or localized macrophage efferocytosis. The key concepts of modern toxicological models are diffusion, metabolism, and clearance; however, these theories may not apply to persistent particulate matter. A physiology-based paradigm that takes into consideration cellular turnover, metabolic demand, and oxidative fragility is crucial to comprehending novel experimental and clinical results, particularly as quantifiable in vivo data is still uncommon and methodologically problematic [10,17,18]. There is presently a profound lack of knowledge regarding long-term tissue retention, which exhibits biological effects throughout tissues, despite rapid advances in detection. Diffusion, metabolism, and clearance are the main principles of current toxicological models; these hypotheses might not hold true for persistent particulate matter [19]. Moreover, there is still a significant lack of knowledge regarding long-term tissue retention because quantitative in vivo findings are still few and technically challenging. In order to enable controlled preclinical designs, innovative and trustworthy methods for the qualitative and quantitative characterization of MNPs in organs have been established [20]. To understand new experimental and clinical findings, a physiology-based paradigm that incorporates cellular turnover, metabolic demand, and oxidative vulnerability is essential. Furthermore, a profound knowledge gap persists regarding long-term tissue retention dynamics, primarily because quantitative in vivo evidence remains scarce and methodologically challenging.

The intracellular fate of internalized particles is not sufficiently taken into consideration by current toxicokinetic models, which assess systemic dispersion through the lenses of particle size, fluid dynamics, and surface charge-mediated translocation. Contrary to these frameworks, the cellular turnover theory emphasizes the programmed lifetime of the host cell as the main kinetic limitation. While the lack of mitotic dilution in low-turnover, high-metabolic tissues trap particles into long-lived cellular structures, creating deep physiological reservoirs, cellular shedding functions as an underappreciated clearance vector in rapidly renewing epithelia. The metabolic stability of MNPs in the intracellular environment of humans provides the physiological justification for this turnover-driven concept. The physical extraction or redistribution of an internalized particle becomes structurally associated with the host cell’s lifecycle and subsequent apoptotic or necrotic disintegration because human cells lack the enzymatic machinery required to break down these carbon backbone structures and because active exocytosis pathways for sub-micron particulates are severely limited.

Here, we present the concept of “bioenergetic accumulation,” which characterizes a scenario in which intracellularly accumulated, non-biodegradable xenobiotics continuously tax the host cell’s metabolism. Particle retention efficiently connects chronic tissue burdens to cellular energy expenditure patterns by forcing persistent energy allocation toward organelle repair, cytoprotective pathways, and lysosomal acidification rather than passive thermodynamic partitioning. After uptake, MNPs acquire a biocorona [21], partially reducing apparent foreignness but maintaining chronic low-grade inflammation [22,23]. Rigid, non-biodegradable MNPs generate a prolonged bioenergetic burden after internalization by interfering with vesicular trafficking, lysosomal activity, and intracellular order. Redox balance, calcium handling, and mitochondrial (membrane potential, ΔΨm) are all impacted, which raises reactive oxygen species (ROS) and decreases adenosine triphosphate (ATP) [24,25]. Over time, cells become metabolically demanding and exhibit extended inflammatory signaling, which compromises tissue integrity.

Recurrent multisystem symptoms encompassing the gastrointestinal, cardiovascular, endocrine, and neurological domains have been suggested based on clinical data from patients with confirmed MNP exposure. These observations offer an exploratory clinical bioenergetic framework. This framework conceptualizes tissue susceptibility to MNPs along three separate biological vectors: resident macrophage capacity for particle phagocytosis and buffering, basal tissue metabolic rate governing bioenergetic maintenance costs, and cellular turnover dynamics dictating whether particles are cleared via cellular renewal or trapped inside deep parenchymal reservoirs (Chicago Cluster), which includes oxidative stress sensitivity, tissue basal metabolic rate (BMRt), and macrophage abundance (Ma) [26]. There is currently no conceptual framework that explains why some organs collect significantly higher particle burdens than others, despite growing evidence of MNP detection in human tissues. Current toxicokinetic models focus on exposure, translocation, and clearance, but these models do not provide sufficient data on intracellular persistence over the long term. In order to explain organ-specific MNP retention and susceptibility, the current study set out to create a physiology-based bioenergetic framework that integrated tissue turnover, macrophage abundance, and metabolic demand. There is presently no conceptual framework that explains why certain organs accumulate noticeably larger MNP burdens than others, despite mounting evidence of MNP detection in human tissues. Exposure, translocation, and clearance are the main topics of current toxicokinetic models; nevertheless, they do not offer enough information on organ-specific sensitivity or long-term intracellular persistence. The current study aimed to develop a physiology-based bioenergetic framework that integrated tissue metabolic requirements, cellular turnover, and macrophage abundance in order to analyze the biological processes governing long-term retention. We looked at patterns of susceptibility to explain observed accumulation profiles and produce testable hypotheses about the biological impacts of long-term MNP exposure using published autopsy-derived tissue concentration data and literature-based physiological measures.

## 2. Materials and Methods

### 2.1. Conceptual Bioenergetic Model

We established the bioenergetic framework, a mechanistic model that describes the kinetics and tissue retention of MNPs in the human body. The fundamental mechanical hypothesis of the framework is that MNPs persist inside the intracellular compartment until host cell death occurs [7,8,9]. This assumption is derived indirectly from the lack of established mammalian exocytosis pathways for large synthetic aggregates and observed post-mortem organ distributions [10,12,17]. We explicitly acknowledge that direct, real-time tracking of this phenomenon in human tissue is currently unfeasible.

### 2.2. Organ/Tissue Selection

The bioenergetic model incorporates 19 organs and tissues selected to capture variation in cellular turnover, metabolic demand, and structural complexity, from rapidly renewing epithelia to long-lived, energy-intensive tissues, such as myocardium and neurons. Organs with high environmental exposure, immune roles, and metabolic constraints were included to reflect real-world vulnerability gradients (Section S6, Supplementary Materials). Three variables define the framework: Ma, indicating immune buffering; tissue BMRt, representing energetic cost of maintenance and intrinsic sensitivity; and the vulnerability index (ISVI), capturing structural fragility under stress.

Three variables define the bioenergetics framework:

(1) Ma, indicating immune buffering;
(2) Tissue BMRt, representing energetic cost of maintenance and intrinsic sensitivity;
(3) The ISVI, capturing structural fragility under stress.

These variables are independent, and no significant correlations (evaluated via non-parametric Spearman rank analysis) are present among them.

These three orthogonal dimensions—immune capacity, energetic demand, and structural vulnerability—allow comparative assessment across tissues. Ordinal scales (1–5) were applied to enable relative, rather than absolute, comparisons in the absence of standardized quantitative metrics. In order to interpret different qualitative histology density and baseline physiological indicators into semi-quantitative orthogonal vectors, the use of 1–5 ordinal scales were tightly operationalized. This method ensures that the model represents intrinsic biological susceptibility rather than just anatomical scale by avoiding the ambiguous scaling effects associated with absolute volumetric differences between different organs. There was actually no subjective distribution of ordinal scores. Each score was mapped onto predetermined categories after being obtained from physiological ranges based on the literature. Heterogeneous datasets provided in various units and procedures across organs were harmonized using the grading system (Sections S1 and S2, Tables S1-T2 and S2-T2).

We developed standardized criteria based on empirical min–max normalization to convert disparate physiological indications into semi-quantitative orthogonal dimensions:

Score = [5 × X_n_]

where X_n_ represents the normalized index value (0.00 to 1.00). The specific operational definitions for scoring assignments are defined as follows:

- Macrophage abundance (Ma): Scored based on steady-state resident density profiles (CD68+ expression). *Tier 1 (Minimal):* Protected zones with highly restricted immune entry (i.e., brain, heart; <10 cells/mm^2^). *Tier 3 (Moderate):* Standard mucosal/interstitial surveillance (10–100 cells/mm^2^). *Tier 5 (Specialized Filter):* Primary systemic filtering networks (i.e., liver, spleen; >500 cells/mm^2^).
- Basal metabolic rate (BMRt): Defined by resting tissue oxygen consumption (VO_2_) and mitochondrial density. *Tier 1:* Minimal metabolic demand (i.e., adipose tissue; <0.5 mL O_2_/min/100 g). *Tier 3:* Standard homeostatic maintenance work (1.0–5.0 mL O_2_/min/100 g). *Tier 5:* Uninterrupted high ATP workload (i.e., brain, myocardium; >10.0 mL O_2_/min/100 g).

We performed a mathematical study to assess the structural stability of our semi-quantitative ranking system. A total of 10,000 Monte Carlo simulations were used to randomly shift each individual parameter score (Ma, BMRt, and ISVI) among the 19 organs by ±1 point. The framework’s relative stratification results are resistant to small score alterations, demonstrated by the upper-tier susceptibility ranking (organs scoring ≥11, such as bone marrow and spleen), which exhibited a retention stability of 94.2%.

### 2.3. Macrophage Content (Ma)

Ma [27,28] was assessed as an index of the intrinsic inflammatory and phagocytic capacity of each tissue. Established histological and immunological data were integrated to characterize the density, distribution, and specialization of resident macrophage populations, including Kupffer cells in the liver, alveolar macrophages in the lung, microglia in the central nervous system, red pulp macrophages in the spleen, and lamina propria macrophages in the intestine, alongside typical homeostatic monocyte infiltration. Tissues were scored on a 1–5 ordinal scale (1 = very low, 5 = very high), where higher values indicate greater baseline potential for particle uptake, cytokine release, and inflammatory amplification (all details are provided in Section S1, Supplementary Materials). The established stereological and immunohistochemical (CD68+/F4/80) tissue density profiles obtained from peer-reviewed human physiological atlases have been employed as a standard for the 1–5 score for Ma. In particular, due to their ubiquitous and well-characterized pan-macrophage glycoprotein surface markers, CD68 (for human tissue profiles) and F4/80 (for supporting rodent mapping) were selected as criteria. This guarantees that baseline, non-activated macrophage densities, rather than briefly produced inflammatory phenotypes, are the basis for our ordinal score. Tier 5 describes specialized, high-density mononuclear phagocyte systems acting as principal systemic filters, whereas Tier 1 indicates minimal resident surveillance (such as blood–brain barrier-protected zones).

### 2.4. Basal Metabolic Rate (BMRt)

Mitochondrial density, oxygen intake, ATP turnover, and basal functional workload are all integrated into BMRt [27,28]. Based on published quantitative measurements of tissue-specific metabolic activity, BMRt was modeled using a 5-point ordinal scale (1 = extremely low, 5 = very high). Each organ’s metabolic reserve and susceptibility are captured by BMRt, a separate bioenergetic axis that is orthogonal to Ma and oxidative stress (OS) sensitivity (Section S2).

### 2.5. Intrinsic Sensitivity and Vulnerability Index (ISVI)

A composite, dimensionless metric entitled the ISVI quantifies a tissue’s innate vulnerability to damage caused by MNPs. The ISVI incorporates three factors: tissue turnover time (Tt) [29,30], Ma [30,31], and baseline cellular index (BCI), a normalized measure of tissue bioenergetic demand [30]. Greater vulnerability is indicated by larger values of the index, which is determined as:

$$ISVI=\frac{BCI}{\left(Ma\times Tt\right)}$$

The biological logic governing this equation is defined as follows: BCI occupies the numerator because tissues with high metabolic maintenance costs are highly sensitive to particle-induced mitochondrial dysfunction. Conversely, Ma and Tt are placed in the denominator because they serve as protective buffering axes. Abundant local macrophages sequester particles within professional phagocytes, protecting sensitive parenchymal cells, while rapid tissue turnover (low Tt value) actively clears internalized contaminants via physiological cell shedding.

The biological hypothesis that circulating local macrophages act as an immune-buffering role is reflected in the placement of Ma in the denominator. Macrophage-rich tissues are better able to sequester particles, control inflammation, and eliminate injured cells, which lessens the effective susceptibility brought on by a prolonged intracellular MNP concentration [31].

The three dimensions—immunological filtering (Ma), basal energetic cost (BMRt), and structural fragility (ISVI)—are verified as statistically orthogonal vectors. Non-parametric Spearman rank correlation analysis across the 19 tissue datasets yielded non-significant collinearity: Ma vs. BMRt (*ρ* = +0.171, *p* = 0.515); Ma vs. ISVI (*ρ*= −0.024, *p* = 0.928); and BMRt vs. ISVI (*ρ* = −0.059, *p* = 0.820). This complete lack of correlation justifies treating them as independent mathematical coordinates of tissue vulnerability.

To facilitate cross-tissue comparability, all variables were normalized to a 0–1 scale (Section S3). The physicochemical characteristics of MNPs are incompatible with classical pharmacokinetics, which is not replicated by this paradigm. Therefore, it provides an energetic fragility and particle retention approximation based on physiology. The three axes—Ma, BMRt, and ISVI—are essentially orthogonal and reflect different biological aspects of immunological buffering, metabolic demand, and structural fragility, according to Spearman correlation analysis [30].

To account for the critical impact of particle characteristics, we introduce a physical scalar modifier (Ω) to modulate the baseline vulnerability score, where:

Score_corrected_ = Score_base_ × Ω

In this case, Ω = f(size, shape, corona). According to published criteria [18,21,32], Ω scales upward (Ω > 1.0) for positively charged surfaces (which accelerate lysosomal destabilization), high aspect ratio fibrous topologies, and sub-micron NPs (<100 nm). Conversely, for large, spherical MPs and NPs with extremely stable biomimetic protein coronas that restrict direct biological contact, Ω scales downward (Ω < 1.0).

### 2.6. Clinical Contextualization of the Vulnerability Model

A cluster of clinical signs and symptoms that are frequently observed in patients who have been exposed to and retained MNPs is referred to as the Chicago Cluster.

We would like to briefly highlight the rationale behind the conceptual framework introduced in this study. In areas where the available evidence is fragmented or heterogeneous, the development of a structured scientific construct can be essential to organize observations, identify recurring patterns, and generate testable hypotheses. The “Chicago Cluster” is presented precisely in this spirit: not as a clinical entity but as a conceptual model aimed at integrating converging clinical and biochemical features that currently lack a unified interpretative structure. Conceptual models have historically played a crucial role in advancing biomedical research, particularly when they precede formal nosological recognition. They provide a provisional but necessary scaffold that allows researchers to articulate mechanistic questions, define inclusion criteria for future studies, and guide the design of validation efforts in independent cohorts. They are explicitly described as hypothesis-generating constructs, open to refinement and external validation, and they are intended to support scientific inquiry rather than clinical classification criteria for future studies and guide the design of validation efforts in independent cohorts. Instead of focusing on single organ dysfunction, they include multisystem involvement, such as cardiovascular, neurological, endocrine–metabolic, and immunological symptoms. Ma, BMRt, and ISVI constitute the bioenergetic framework in this study. The cluster is used solely as an exploratory external clinical comparator and should not be interpreted as independent validation of the proposed model. C_e_ represents cerebral, H is for hematological, I is for intestinal, Ca is for cardiovascular, G is for gynecological, O is for oncological, C_o_ is for connective, L is for liver, U is for urological, S is for sensory, T is for thyroid, E is for endocrine, and R is for respiratory. Section S4 provides a detailed classification of organs and tissues by cluster and pathophysiology attribution.

## 3. Results

Ma content, BMRt, and ISVI sensitivity of 19 organ/tissues are summarized in Table 2.

Due to tissue metabolic demand, macrophage abundance, and structural vulnerability corresponding to different physiological factors, the observed non-collinearity is physiologically justifiable. In particular, tissues with sluggish turnover may exhibit either high or low energy demand, but high metabolic activity does not always require a high resident macrophage density. As a result, rather than capturing duplicate elements of tissue physiology, the three variables record complementary elements.

A score variance greater than one point does not denote direct statistical significance but rather indicates a categorical shift across distinct susceptibility brackets, distinguishing tissues with high immunometabolic risk from those with slower accumulation dynamics. Consistent with established physiology, the heart, brain, liver, and highly active epithelial tissues occupy the upper range of basal metabolic demand. This is due to its high metabolic throughput, significant cellular turnover, and ongoing immunological activation. As a result, it is very sensitive to MNPs and is an excellent option for early biomarker identification. Organs with a score of 11, such as the spleen, lung, and intestinal epithelium, are considered early reporters of MNP exposure because they are physiological hubs with immunological, respiratory, and nutritional roles, respectively. Parts of the testes and other organs with a score of 10 have moderate turnover, metabolic activity, and vulnerability to oxidative stress. The vascular endothelium, which similarly obtained a score of 10, is a major interface for MNP translocation and accumulation due to its direct contact with blood. According to clinical findings, MNPs in carotid atherosclerotic plaques are associated with higher cardiovascular risk, plaque destabilization, and enhanced inflammatory activation [29], which is in accordance with bioenergetic predictions [33].

Tissues with a score of less than 10 exhibit slower Ma turnover and lower BMRt, maintaining adaptive capability and lowering systemic risk. The brain is one of the least reactive tissues, indicating a solid immunological, metabolic, and structural response that postpones MNP-induced neurological consequences and is consistent with the requirement for long-term exposure until neurotoxicity occurs.

### 3.1. The Chicago Cluster in Relation to the Bioenergetic Framework

For each organ, the 26 signs and symptoms associated with 40 acute or chronic diseases attributed to microplastic-induced syndrome (MI-Syn) were recorded [26]. This analysis tested whether tissues predicted as highly vulnerable by Ma, BMRt, and ISVI correspond to clinically affected systems. Alignment between bioenergetic vulnerability and observed manifestations provides an external clinical framework of MNP-related pathophysiology (Table 3).

Despite indicating multisystem involvement, the 26 symptoms and signs constitute a logical biological pattern. Fatigue (symptom 12), which predominantly affects the brain; abdominal discomfort (symptom 1), which is associated with the gastrointestinal tract; high ESR/CRP (symptom 18), which reflects persistent low-grade inflammation; and ultrasonography abnormalities (symptom 20), which indicate tissue destruction, are common signs [26].

Clinical characteristics that regularly correlate with organ and tissue dysfunctions are determined by the Chicago Cluster. The bioenergetic framework’s predictions are supported by an analysis of the 19 significant tissues. The most clinically impacted organs are those with high macrophage abundance, BMRt, and ISVI, which demonstrated an exploratory correlation between predicted tissue vulnerability and clinical symptom presentation. These patterns suggest statistical associations that require prospective verification before any causal inferences can be drawn [26].

Within our model, the brain has a distinct compartmental character. Its intrinsic vulnerability index is highest (ISVI = 5); however, its low cumulative score (7) indicates limited baseline entrance and low macrophage density (Ma = 1). This resolves an apparent paradox: the brain functions as a deep kinetic repository that accumulates slowly and is shielded by the blood–brain barrier. Nevertheless, once translocation occurs, in effect, it has little structural or mitotic capacity to buffer or dilute particle burdens, rendering it extremely vulnerable to long-term neurotoxic friction.

We emphasize that while including headache, fatigue, and abdominal pain are very non-specific and common to many diseases, the Chicago Cluster does not provide independent validation of our paradigm. Instead, the cluster is used as an exploratory, hypothesis-generating clinical reference model to compare reported symptom patterns in a highly exposed population with tissue vulnerability scores.

### 3.2. Hypothesis-Generating Bioenergetic Damage Panel (Three-Marker Framework)

The bioenergetic framework was employed to identify laboratory markers of early MI-Syn. Three blood-based variables—oxidative load, perfusion mismatch, cytolytic stress, and myocardial vulnerability—capture early systemic distress in organs scoring ≥10 in the Table 4 matrix. In high-throughput, oxygen-dependent tissues, such as the heart, neurons, liver, and alveolar epithelium, plasma lactate provides an early indicator of bioenergetic strain due to decreased oxidative phosphorylation and oxygen delivery mismatch [34,35]. In high-turnover organs, serum lactate dehydrogenase (LDH) integrates glycolytic–mitochondrial flow and structural renewal, indicating cytosolic stress. As a sensitive indicator of metabolic strain rather than lipid metabolism, LDH is released in response to even slight changes in perfusion, redox balance, or cellular integrity [36,37]. When combined, these markers provide a straightforward yet effective panel for the early detection of organ-level bioenergetic abnormalities when recognizable clinical symptoms appear. High-throughput, perfusion-dependent organs are disproportionately affected by high-sensitivity CRP (hsCRP), a sign of systemic inflammatory response [38]. When combined, these three criteria provide an essential panel for the early identification of bioenergetic disruption at the organ level prior to obvious clinical symptoms. The threshold values of the three variables are reported in Table 4; details are reported in Tables S5-T1–S5-T4.

This circulating triad–lactate, LDH, and hsCRP must not be interpreted as a diagnostic or clinical tool for MNP exposure, given their highly non-specific nature and elevation across numerous common ischemic, infectious, and neoplastic diseases. Instead, they are proposed strictly as an exploratory metabolic signature to track systemic bioenergetic stress in controlled study settings. A systemic shift towards quicker anaerobic glycolysis is reflected in the corresponding, subclinical rise of plasma lactate and serum LDH, while hsCRP sets the baseline of sterile, macrophage-driven chronic inflammation produced by the persistent intracellular particle concentration. This particular biochemical triad provides a unique diagnostic signature of microplastic-induced syndrome (MI-Syn; hypothesis-generating clinical classification) prior to the onset of macrovascular tissue necrosis, capturing the chronic, low-grade metabolic friction caused by ongoing lysosomal destabilization and mitochondrial ΔΨm disruption across high-turnover tissues [26,39].

We recognize that lactate, LDH, and hsCRP are non-specific indicators that may increase in response to acute infection, tissue ischemia, trauma, cancer, and systemic inflammatory diseases. Therefore, rather than representing a diagnostic indicator of MNP exposure, their suggested function should be considered as a bioenergetic signature that generates hypotheses. Clinical history, exposure evaluation, the temporal persistence of biomarker increase, and the exclusion of acute inflammatory, infectious, and ischemic diseases should all be incorporated into differential diagnosis. MNP-associated bioenergetic strain is thought to appear as a chronic low-grade metabolic pattern, in contrast to acute ischemic syndromes, which usually result in sudden and noticeable biomarker increases [40]. It is important to emphasize that lactate, LDH, and hsCRP are not specific to MNP-driven diseases; rather, they are conventional non-specific indicators of systemic stress. Within this paradigm, their use can only be exploratory and hypothesis-generating; clinical application would absolutely be required to exclude typical acute inflammatory, infectious, or ischemic disorders beforehand.

## 4. Discussion

MNPs have toxicological behaviour that significantly differs from the conventional principles of absorption, distribution, metabolism, and excretion (ADME). Diffusion and metabolic clearance cannot forecast their distribution since the human body lacks the biochemical mechanisms required to digest synthetic polymers as biological macromolecules. After internalization, MNPs cause cumulative harm by remaining inside host cells until cell death. This retention is irreversible and eventually leads to cellular dysfunction and death, even at low-grade inflammatory levels.

### 4.1. Bioenergetic Vulnerability as the Key Determinant of Toxicity

Organ-specific bioenergetic vulnerability is illustrated in Table 2. Bone marrow has the highest ranking (12 points) due to its refractory metabolic recycling and ongoing innate immune activation in response to MNPs. The spleen, lung, and intestinal epithelium are among the organs with a score of 11 that constitute the functional axis of hematopoietic turnover, immune surveillance, and environmental interface [41]. The lung and intestinal epithelium are primarily subjected to mechanical, chemical, and biochemical attacks, while the spleen enhances marrow function through selective filtration and maturation. Tissues with a score of 10 belong to an intermediate bioenergetic load range that integrates significant ISVI, considerable BMRt, and Ma [41]. This combination generates a continuous energy burden that is more sustained than in low-turnover organs, such as the brain or adipose tissue, but it is less extreme than in high-turnover tissues. These organs often act as physiological interfaces, whether they are vascular, metabolic, endocrine, or reproductive, necessitating constant function in a variety of scenarios. These organs include the liver, which integrates detoxification, protein synthesis, and metabolic buffering; the kidney, which maintains solute transport and filtration; the pancreas, which regulates endocrine and exocrine output; the vascular endothelium, which is exposed to shear stress and inflammatory signaling; the placenta, which regulates nutrient exchange and immunological tolerance; and the testes, which carry out endocrine activity with spermatogenesis. These tissues are exposed to cumulative subclinical stress, such as long-term exposure to MNPs, which may gradually exhaust functional reserves despite their functional variability [41].

### 4.2. Tissue Distribution Hypothesized to Be Modulated by Cellular Turnover

The application of this bioenergetic model’s application suggests that MNP distribution dynamics may be significantly modulated by cellular turnover. According to autopsy outcomes, intermediate-turnover organs (i.e., liver, kidney, lung, endothelium) retain a number of particles over months, whereas rapid-turnover tissues (i.e., intestine, epidermis, blood) exhibit minimal retention [42,43]. Heart, brain, and adipose tissues are low-turnover tissues that act as long-term reservoirs. The distribution of MNPs reflects host cell lifecycles rather than diffusion, which clarifies the reason metabolically active, minimally regenerating organs are disproportionately vulnerable, according to model predictions and post-mortem studies [42,43].

By differentiating between accumulation dynamics and bioenergetic vulnerability, this operational mechanism overcomes an apparent dilemma within our model hierarchy. In post-mortem investigations, rapid-turnover tissues, such as the bone marrow and intestinal epithelium, exhibit minimal long-term particle retention because contaminated cells are constantly eliminated during regular renewal cycles. Nevertheless, in order to maintain tissue homeostasis under persistent MNP-induced cytotoxicity, this continuous clearance places an enormous, compounding energy and BMRt burden on the stem cell niche, leading to their highest susceptibility scores. Low-turnover tissues, such as the brain and heart, conversely, function as deep, passive reservoirs where a lack of replication results in a steady, accumulated intracellular concentration, creating a distinct, time-dependent pathway to chronic organ failure.

### 4.3. Post-Mortem Data

The proposed bioenergetic framework shows exploratory alignment with preliminary post-mortem observations [12,44]. We acknowledge that these human tissue datasets remain highly limited, structurally heterogeneous, and constrained by potential analytical contamination. They should be interpreted as qualitative, preliminary observations rather than definitive validation of turnover-driven kinetics. Contrary to perfusion-driven pharmacokinetics, low-turnover organs (brain, kidney, and liver) gradually retain MNPs along a gradient of brain > kidney > liver [44,45]. The hypothesis that MNPs accumulate in metabolically active cells and are only released at cell death is supported by the findings. Blood represents dynamic cellular loss, whereas slow-turnover tissues function as deep, slowly equilibrating storage capacity. This proposed two-compartment toxicokinetic framework remains a highly speculative theoretical construct (bioenergetically limited deep compartment long-lived tissues with very slow clearance and a tiny and quickly exchanging central compartment of blood) [12,44,45,46]. It is presented here not as a validated mathematical reality but as a conceptual model to help design future prospective animal tracking studies and quantify deep vs. central clearance velocities. A non-linear, two-compartment toxicokinetic paradigm is theoretically consistent with this distribution. Due to continuous cellular clearance and mechanical shear stress, the central compartment (blood/plasma) exhibits rapid, transitory dynamics with poor homeostatic retention (1–100 ng/mL). Conversely, the deep compartment is composed of highly vascularized, metabolically active organs with slow cellular renewal (such as the heart and liver parenchyma), where the inward clearance velocity (Cl in) is significantly greater than the outward clearance velocity (Cl out), which is rate-limited by the host cell’s programmatic lifespan (Tt) [12,44,45].

### 4.4. Clinical Implications: The Chicago Cluster and Bioenergetic Framework

A plausible interpretive model for the multisystem toxicity of MNPs is provided by combining the Chicago Cluster with an organ-specific bioenergetic framework. A subset of 40 disorders with a common clinical symptom profile was determined from the identification of 26 recurrent symptoms in the MNP toxicological literature and their application to 235 diseases representing 21 pathological categories. This combination indicates that exposure to MNPs induces a more comprehensive syndrome-level phenotype, here referred to as MI-Syn, rather than distinct organ-specific diseases [22]. This framework’s Bayesian classification method constitutes one of its key strengths. A posterior probability of ≥95% for MNP involvement is associated with the presence of at least eight elements, including two exposure indicators and six sign/symptoms from the Chicago Cluster, when symptom frequency and environmental exposure factors are integrated. This threshold provides a statistically reliable and reproducible criterion that minimizes over-attribution by representing both the high discriminative value of the identified symptoms and the low prior probability of confirmed MNP-related medical conditions [47,48]. The Chicago Cluster has not yet undergone independent external validation and, therefore, should be regarded as a preliminary clinical framework rather than a recognized disease entity.

Biological plausibility is further demonstrated by mapping the 40 MI-Syn illnesses across 19 organs and tissues. Highly fragile organs are disproportionately represented, according to the bioenergetic fragility index, which is generated from Ma, tissue-specific BMRt, and the ISVI. This implies that tissues with high immunometabolic requirements or little energy storage are more susceptible. Clinically, this degree of sensitivity is consistent with recurrent symptoms such as dyspepsia, persistent fatigue, and increased inflammatory markers. Dyspepsia is associated with gastrointestinal fragility, chronic fatigue is symptomatic of reduced mitochondrial efficiency, and inflammatory indicators are predictive of macrophage activity [47,48]. Early alterations to hsCRP, lactate, and LDH also make it feasible to identify subclinical metabolic disorders and provide prompt therapy.

The ≥95% posterior probability threshold for MNP-driven systemic involvement was calculated utilizing a discrete Bayesian updating algorithm:

$$P\left(Mi-Syn\;÷Symptoms\right)=\;\frac{P\left(Symptoms\;÷MI-Syn\right)\times \;P\;(MI-Syn)}{P\;(Symptoms)}$$

The following explicit assumptions are used to parameterize the exploratory posterior probability calculation. We construct a conservative prior probability *P(MI-Syn)* = 0.01, which reflects the condition’s unconfirmed state. Based on previous environmental cohort studies, the true positive probability for displaying ≥6 distinct cluster symptoms coupled with ≥2 exposure vectors is established at *P(Symptoms⋅MI-Syn)* = 0.85 [26]. Due to the great mathematical specificity required by the simultaneous existence of eight different criteria, the false positive background rate in the general unexposed population is limited to *P(Symptoms⋅Control)* = 0.0004. The exploratory posterior value obtained by applying these settings via sequential Bayesian updating is:

$$P\frac{MI-Syn}{Symptoms}=\frac{0.85\;\times \;0.01}{\left(0.85\;\times \;0.01\right)+(0.0004\;\times \;0.99)}=0.955$$

This value is intended strictly as a hypothesis-generating threshold for clinical trial design.

The clustering of ≥6 clinical signs/symptoms and ≥2 documented environmental exposure vectors provides the mathematical weight required for crossing the critical significance threshold, minimizing false positive syndromic attributions, given the conservative prior probability *(P(MI-Syn))* assigned due to the novelty of the hypothesis-generating clinical classification.

### 4.5. A Unified Paradigm for Microplastic Toxicity

The findings provide credibility to the hypothesis that chronic MNP toxicity is caused by the combination of bioenergetic fragility and particle accumulation rather than from exposure alone. This methodology highlights the significance of incorporating MNP burden, mitochondrial demand, and tissue-specific energy resilience into toxicological models and provides a mechanistic explanation for the occurrence of clinical symptoms in exposed populations. Notably, three readily available early diagnostic indicators—lactate, LDH, and hsCRP—have been established based on a wide range of medical conditions identified among the 19 organs and tissues selected for consideration. Nevertheless, these parameters have to be confirmed in controlled studies. Future studies should use multi-omic procedures, longitudinal designs, and direct indicators of cellular turnover. In summary, metabolic demand rather than passive diffusion processes regulates the distribution of MNPs. To thoroughly validate these turnover-driven dynamics, it would be essential to use standardized measurement tools in controlled preclinical models, such as the validation techniques suggested [20].

### 4.6. Comparison with Extant Experimental Exposure Data

We compared our organ scores with current rodent exposure models in order to evaluate the validity of our qualitative method. The spleen, liver, and small intestine margins consistently exhibit the largest mass-normalized accumulation in controlled mouse studies, including the oral intake of polystyrene (PS) beads [13,14]. This is congruent with our high susceptibility ranking for these major filtration and mucosal interfaces (scoring 11 and 10). Additionally, our model’s attribution of the greatest susceptibility score (12) to bone marrow tissue is directly empirically supported by recent evidence revealing NP translocation into bone marrow in mice habitats and subsequent hematopoietic stem cell stress [7,41].

## 5. Putative Limitations

Several limitations should be considered when interpreting these findings. First, the integration of heterogeneous datasets—spanning different analytical platforms, study designs, and reporting standards—introduces variability that may affect cross-study comparability and quantitative precision. Second, the proposed framework is grounded in biologically plausible mechanisms but requires validation in larger and more diverse populations to establish generalizability. Third, key aspects of long-term exposure dynamics and inter-organ variability remain incompletely resolved at the molecular level, particularly with respect to degradation pathways and tissue-specific clearance. Finally, the predominance of cross-sectional data limits causal inference, underscoring the need for longitudinal and interventional studies to define temporal relationships and confirm mechanistic links. Additionally, MNPs are considered as a semi-homogeneous particulate phase in the current iterations of the bioenergetic framework. The physicochemical heterogeneity of the polymers, particularly the aspect ratio, surface charge, diameter (nano vs. micro), and the dynamic composition of the protein corona, significantly impact toxicokinetics in vivo. These characteristics must be included in the inherent sensitivity equations as independent scalar modifiers in subsequent empirical calibrations. MNPs are considered a semi-homogeneous particulate phase in this current model. Particle size, aspect ratio, polymer composition, surface charge, crystallinity, and biocorona formation are examples of physicochemical modifiers that should be included in future revisions. These characteristics might be used as scalar adjustment variables that affect intracellular persistence, bioenergetic burden, and retention efficiency [18,32].

Additionally, this study’s dependence on semi-quantitative ordinal indicators generated from the literature offers a systemic restriction (1–5). The framework functions as a hypothesis-generating tool rather than a formal statistical validation since the variables (Ma, BMRt, and cellular turnover parameters) are constructed from descriptive physiological facts. Since the Chicago Cluster’s clinical indicators are also based on retrospective reports of exposed populations, we also recognize the possibility of cross-interpretive circularity. For the aim of guiding future empirical, molecularly resolved tracking studies, these models must be strictly understood as conceptual starting points.

## 6. Conclusions

Current understanding of MNP biology is constrained by the absence of standardized quantification and polymer-resolved analyses, limiting definitive causal attribution. Nevertheless, the present study advances a conceptual framework in which MNP accumulation is governed not by passive diffusion alone but by the interplay between cellular turnover and bioenergetic demand. By comparing our conceptual model with preliminary, qualitative human post-mortem observations, we outline a theoretical framework that presents the intracellular retention cellular renewal axis as a proposed conceptual hypothesis that may explain MNP persistence dynamics. This turnover-driven paradigm provides a coherent explanation for the preferential accumulation of MNPs in metabolically active and immunologically dynamic tissues and links their presence to convergent pathways of oxidative stress, energetic imbalance, and chronic inflammation. These findings position bioenergetic disruption as a measurable interface between environmental exposure and human pathology. The emergence of a minimal circulating signature—lactate, LDH, and high-sensitivity hsCRP—suggests that systemic consequences of MNP burden may be detectable before overt tissue dysfunction, although rigorous prospective validation is required. We emphasize that turnover-driven dynamics need to be rigorously validated in future exposure models, as existing human observational data are still indirect.

More broadly, this study reframes MNPs from passive contaminants to dynamic participants in human physiology, whose persistence arises from fundamental cellular processes. Resolving their long-term impact will require longitudinal human studies, controlled exposure systems, and molecularly resolved tracking approaches capable of capturing intracellular fate, degradation, and clearance [20]. Establishing such frameworks will be essential for translating mechanistic insight into predictive toxicology and effective intervention strategies.

## Figures and Tables

**Table 1:** Microplastic levels in blood vs. tissue.

Species/Model	MNPs Type/Size	Circulating Matrix Concentration	Target Organ Concentration	Calculated Tissue-to-Blood Gradient Ratio	Reference
Human (autopsy/clinical)	MPs	Blood: 1.84 ± 0.28 μg/mL	Liver: 10–1500 ng/g; lung: 10–10,000 ng/g	10–100× accumulation in solid parenchymal architectures	[7,8,11]
Human (autopsy)	NPs	Plasma	Brain (frontal cortex): 4.8 mg/g tissue	1000× highly variable deep compartmentalization	[6,8,12]
Rodent (oral/inhalation)	PS-NPs (50 nm)	Blood: 0.5–5.0 ng/mL (transient)	Spleen: 50–100 μg/g; liver: 20–80 μg/g	100–1000× driven by mononuclear phagocyte filtration	[11,13,14,15]
Livestock (bovine/ovine)	mixed MPs (10–50 μm)	Whole blood: 5–30 ng/mL	Edible muscular tissue: 10–150 ng/g; kidney cortex: 50–400 ng/g	2–20× gradient emphasizing filtration boundaries	[16]

**Table 2:** Cumulative scores for organ and tissue susceptibility based on Ma, BMRt, and ISVI parameters.

Organ/Tissue	Ma	BMRt	ISVI	Total
Bone marrow	4	4	3	12
Spleen	5	3	3	11
Lung (alveolar epithelium)	4	3	4	11
Intestinal epithelium	3	4	3	11
Liver	4	4	3	10
Kidney	3	3–4	4	10
Pancreas	3	3	4	10
Vascular endothelium	3	4	3	10
Placenta	3	4	3	10
Testis	2	3	5	10
Skin (epidermis)	3	4	2	9
Heart	2	5	5	9
Uterus	3	3	3	9
Ovaries	2	2	4	8
Thyroid	2	3	3	8
Brain	1	5	5	7
Adipose tissue	3	2	2	7

The Spearman correlations indicate that the three variables were completely independent: Ma vs. BMRt = *ρ* + 0.171 (*p* = 0.515); Ma vs. ISVI = *ρ* − 0.024 (*p* = 0.928); BMRt vs. ISVI = *ρ* − 0.059 (*p* = 0.820). For details, see Sections S1–S3.

**Table 3:** Organ/tissue—associated sign/symptoms (Chicago Cluster).

Organ/Tissue	Associated Sign/Symptom	Chicago Cluster Number ^a^
Liver	Irregular bowel habits, persistent fatigue, persistent headache, liver enzyme modifications, elevated ESR/CRP, unexpected fever, dyslipidemia, ultrasound changes	1–12–13–17–18–22– 23–20
Spleen	Irregular bowel habits, persistent fatigue, blood disorders, elevated ESR/CRP	1–12–18–25
Lung	Irregular bowel habits, cough/dyspena, elevated ESR/CRP, unexpected fever, persistent fatigue, persistent headache, ultrasound changes	1–7–12–13–17–18–20
Bone marrow	Irregular bowel habits, persistent fatigue, elevated CRP/ESR, lymphadenopathy, unexpected fever, blood disorders	1–12–17–18–19–25
Gut epithelium	Bloating/irregular bowel habits, abdominal pain, dyspeptic symptoms, blood in stools, persistent fatigue, persistent headache, elevated ESR/CRP, unexpected fever	1–2–3–4–12–13–17–18
Skin/epidermis	Dermatitis, allergies, sensorial disorders, elevated ESR/CRP	5–6–15–18
Kidney	Irregular bowel habits, persistent fatigue, persistent headache, creatinine modification, elevated ESR/CRP, unexpected fever, ultrasound changes	1–12–13–17–18–20– 24
Heart	Irregular bowel habits, persistent fatigue, persistent headache, hypertension, swelling, elevated ESR/CRP, unexpected fever, ultrasound modifications	1–12–13–16–17–18–19 –20
Brain	Irregular bowel habits, behavioral changes, cognitive changes, persistent headache, persistent fatigue, visual disturbances, sensorial disorders, elevated ESR/CRP, unexpected fever, ultrasound changes	1–10–11–12–13–14–15 –17–18–20
Pancreas	Irregular bowel habits, behavioral changes, persistent headache, persistent fatigue, elevated ESR/CRP, pancreatic enzyme modification, ultrasound changes, unexpected fever, fasting blood glucose increase	1–10–12–13–17–18–20 –21–26
Skeletal muscles	Sensorial disorders, ultrasound changes	12–15–20
Testis	Changes in sexuality, hypertension, elevated ESR/CRP, ultrasound changes	9–16–18–20
Uterus	Bloating, abdominal pain, behavioral changes, menstrual changes, change in sexuality, unexpected fever, persistent headache, persistent fatigue, elevated ESR/CRP, ultrasound changes	1–2–8–9–10–12–13–17 –18–20
Ovaries	Bloating, abdominal pain, behavioral changes, menstrual changes, change in sexuality, unexpected fever, persistent headache, elevated ESR/CRP, ultrasound changes	1–2–8–9–10–12–13– 17–18–20
Thyroid	Irregular bowel habits, menstrual changes, change in sexuality, persistent fatigue, behavioral changes	1–9–10–12–13
Vascular endothelium	Hypertension, persistent headache, lymphedema, elevated ESR/CRP, venous insufficiency, ultrasound changes	12–13–16–18–19–20
Placenta	Bloating, irregular bowel habits, abdominal pain, unexpected fever, elevated ESR/CRP, ultrasound changes	1–2–12–17–18–20
Bones	Persistent fatigue, ultrasound modifications	12–20
Abdominal fat	Bloating, abdominal pain, dyslipidemia, ultrasound changes	1–2–18–20

^a^ = For the meaning of sign/symptoms numbers, see Table S4-T1.

**Table 4:** Clinical pathological thresholds for MNPs.

Biomarker	Unit	Normal Range	Pathological Threshold	Clinical Notes
Lactate	mmol/L	0.5–2.0	>2.0	>2.0 mmol/L suggest hypoperfusion; >4.0 mmol/L with acidosis indicates critical illness
LDH	U/L	120–250 (method-dependent)	>250	Non-specific marker of tissue injury; >250 U/L strongly suggests acute damage
hsCRP	mg/L	<1.0	>3.0	>3.0 mg/L is the key threshold for cardiovascular risk stratification

## Data Availability

The original contributions presented in this study are included in the article/Supplementary Material. Further inquiries can be directed to the corresponding author.

## Supplementary Materials

The following supporting information can be downloaded at: https://www.mdpi.com/article/10.3390/toxics14070603/s1, Section S1: Macrophages content (Ma); Section S2: Basal Metabolic Rate (BMRt); Section S3: Intrinsic Sensitivity and Vulnerability Index (ISVI); Section S4: Chicago Cluster; Section S5: Bioenergetic panel index; Section S6: Bioenergetic specific organ details; Section S7: Bioenergetic Damage Panel (3 marker framework); Table S1-T1. Scores (1–5) of the macrophages content in the 19 organ and tissues; Table S1-T2. Macrophage Scoring Criteria; Table S2-T1. Tissue Basal Metabolic Rate (BMRt) Table—19 Organs/Tissues; Table S2-T2. BMRt Scoring Criteria; Table S3-T1. Components of the Intrinsic Sensitivity and Vulnerability Index (ISVI) Across 19 Organs; Table S3-T2 Values of ISVI (total) and references; Table S4-T1. Chicago Cluster signs and symptoms; Table S4-T2. Functional Organ/Tissue Clusters and Corresponding Clinical Domain; Table S4-T3. Unified Table—Diseases and Symptoms considerd as part of MIC-Syn; Table S4-T4. Sygn and Symptoms frequency; Table S4-T5. Final Integrated Bioenergetic sensitivity (Descending Total Score); Table S5-T1. Clinical Pathological Thresholds for MNP; Table S5-T2. MIC Syn Bioenergetic Diagnostic Table; Table S5-T3. MIC Syn Bioenergetic Risk Classification; Table S5-T4. Optional: Severity Index (only for MIC Syn–positive subjects); Table S6. Single variables score and total score of framework evaluation.

## Abbreviations

The following abbreviations are used in this manuscript:

ADME	Absorption, Distribution, Metabolism, and Excretion
ATP	Adenosine Triphosphate
BCI	Baseline Cellular Index
BMRt	Basal Metabolic Rate
ΔΨm	Membrane Potential
hsCRP	C-Reactive Protein
ISVI	Intrinsic Sensitivity and Vulnerability Index
LDH	Lactate Dehydrogenase
Ma	Macrophage Abundance
MNPs	Micro-nanoplastics
MI-Syn	Microplastic-induced Syndrome
OS	Oxidative Stress
PS	Polystyrene
Tt	Turnover Time
