# Supplementary material for: A Bioenergetic Framework for Microplastic Accumulation in Human Tissues: A Cellular Turnover Hypothesis
Source: Toxics. 2026 Jul 10;14(7):603. doi: 10.3390/toxics14070603 (PMC13417828; doi:10.3390/toxics14070603)
Supplement: Supplementary file 1 [file toxics-14-00603-s001.zip › toxics-4375020-supplementary.pdf]

Supplementary Materials

A Bioenergetic Framework for Microplastic Accumulation in Human Tissues: A Cellular Turnover Hypothesis

Umberto Cornelli [1,*], Giuseppe Zanoni [2] and Claudio Casella [2]

1 Department of Molecular Pharmacology and Therapeutics, School of Medicine, Loyola University, 2160 1st Ave, Maywood, IL 60660, USA

2 Department of Chemistry, University of Pavia, Viale Taramelli 12, 27100 Pavia, Italy; gz@unipv.it (G.Z.); claudio.casella01@universitadipavia.it (C.C.)

* Correspondence: ucornelli@gmail.com

Index:

Argument	Section
Summary	
Macrophages content (Ma)	S1
Basal Metabolic Rate (BMRt)	S2
Intrinsic Sensitivity and Vulnerability Index (ISVI)	S3
Chicago Cluster	S4
Bioenergetic panel index	S5
Bioenergetic specific organ details	S6
Bioenergetic Damage Panel (3-marker framework)	S7

Summary

The inherent characteristics of the tissues that micro-nanoplastics (MNPs) contaminate determine the risk these are in relation to humans. Every organ or tissue has a different degree of susceptibility, which can be thought of as its bioenergetic reactivity, which determines how resistant it is to damage caused by MNPs. The study has four goals:

1. To define a set of independent, non-collinear variables that capture the bioenergetic reactivity of 19 major human organs/tissues.
2. To quantify the bioenergetic reactivity of 19 organs/tissues affected by MNP contamination and identify those with the highest susceptibility.
3. To identify possible pathophysiological crossings by comparing the Chicago Cluster's descriptive categories with organ-level bioenergetic rankings.
4. To determine whether simple laboratory tests can be identified that allow early detection of MNP-induced damage, based on the integration of organ-level susceptibility and the Chicago Cluster.

To identify relevant literature for each component, structured searches were performed in PubMed, Web of Science, and Scopus using combinations of criteria pertaining to MNPs, organ-specific physiology, bioenergetics, and clinical outcomes. To obtain additional information, backward and forward citation monitoring were also used. Peer-reviewed articles and reliable reports were the only ones included. Search algorithms were customised for each chapter to guarantee thorough coverage of organ-specific processes, toxicological pathways, and clinical manifestations associated with MNP exposure.

Supplementary S1

Macrophage content (Ma)

The density of resident macrophages under physiological conditions and the propensity for macrophage infiltration were represented by the macrophage abundance score, which ranged from 1 to 5. Kupffer cells in the liver, alveolar macrophages in the lung, microglia in the brain, and red pulp macrophages in the spleen are an instance of tissue resident macrophage populations that were described using recognised histological and immunological atlases.

The relative inflammatory amplification potential of each tissue in response to particle intake is represented in macrophage scores:

Score	Interpretation
1	Very low content of macropage)
2	Low number of macrophage
3	Medium number of macrophages
4	High number of macrophages
5	Very high number of macrophages

Table S1-T1 reports the scores of the 19 organ/tissues and relative references.

Table S1-T1. Scores (1-5) of the macrophages content in the 19 organ and tissues

Rank	Organ / Tissue	Score 1-5	Key References
1	Liver	5	Krenkel, O., & Tacke, F. Liver macrophages in tissue homeostasis and disease. *Nature Reviews Immunology* **17(5)**, 306–321 (2017). https://doi.org/10.1038/nri.2017.11
2	Spleen	4	den Haan, J. M. M., Kraal, G., & Bevan, M. J. Cutting edge: Macrophages in the spleen. *Journal of Immunology* **188(9)**, 4441–4445 (2012). https://doi.org/10.4049/jimmunol.1200165
3	Lung	4	Hussell, T., & Bell, T. J. Alveolar macrophages: Plasticity in a tissue-specific context. *Nature Reviews Immunology* **14(2)**, 81–93 (2014). https://doi.org/10.1038/nri3600
4	Bone marrow	4	Chow, A., Huggins, M., Ahmed, J., Hashimoto, D., Lucas, D., Kunisaki, Y., Pinho, S., Leboeuf, M., Noizat, C., van Rooijen, N., et al. Bone marrow macrophages maintain hematopoietic stem cell niches**.** *Nature* **475**(7357), 214–218 (2011). https://doi.org/10.1038/nature10238
5	Intestinal epithelium	3	Bain, C. C., & Mowat, A. M. The monocyte–macrophage axis in the intestine. *Cellular Immunology* **291**(1–2), 41–48 (2014). https://doi.org/10.1016/j.cellimm.2014.05.010
6	Skin	2	Malissen, B., Tamoutounour, S., & Henri, S. Macrophages and dendritic cells of the skin. *Nature Reviews Immunology* **14(6**), 417–428 (2014). https://doi.org/10.1038/nri3683
7	Kidney	1	Bergmann, M., Mützel, S., Primpke, S., Tekman, M. B., Trachsel, J., & Gerdts, G. *White and wonderful? Microplastics prevail in snow from the Alps to the Arctic. Science* **363,** (6432), 1100–1102 (2019). https://doi.org/10.1126/science.aav6839
8	Heart	1	Epelman, S., Lavine, K. J., Beaudin, A. E., Sojka, D. K., Carrero, J. A., Calderon, B., et al. Embryonic and adult-derived resident cardiac macrophages**.** *Immunity* **40(1)**, 91–104 (2014). https://doi.org/10.1016/j.immuni.2013.11.019
9	Brain	3	Silvin, A., Qian, J., & Ginhoux, F. Brain macrophage development, diversity and dysregulation in health and disease. *Cellular & Molecular Immunology* (2023). https://doi.org/10.1038/s41423-023-01064-6
10	Pancreas	1	Calderon, B., et al. The pancreas anatomy of macrophages. *PNAS* **112(35)**, E4256–E4265 (2015). https://doi.org/10.1073/pnas.1513509112
11	Skeletal muscle	1	Tidball, J. G. Regulation of muscle growth and regeneration by the immune system. *Nature Reviews Immunology* **17(3)**, 165–178 (2017). https://doi.org/10.1038/nri.2016.150
12	Testis	1	DeFalco, T., Bhattacharya, I., Williams, A. V., Sams, D. M., & Capel, B. Macrophages in the testis**.** *Development,* **142**(14), 2582–2592 (2015). https://doi.org/10.1242/dev.126706
13	Uterus	4	King, A. E., Critchley, H. O. D., & Kelly, R. W. Macrophages in the human endometrium and their role in tissue remodelling. *Journal of Reproductive Immunology,* **88(2)**, 141–148 (2010). https://doi.org/10.1016/j.jri.2010.01.005
14	Ovaries	3	Wu, R., Van der Hoek, K. H., Ryan, N. K., Norman, R. J., & Robker, R. L**.** Macrophages in ovarian function. *Reproduction,* **127(6)**, 791–801 (2004). https://doi.org/10.1530/rep.1.00180
15	Vascular Endothelium	2	Gimbrone, M. A., & García-Cardeña, G. Endothelial cell dysfunction and the pathobiology of atherosclerosis. *Circulation Research* **118(4)**, 620–636 (2016). https://doi.org/10.1161/CIRCRESAHA.115.306301
16	Placenta	4	Burton, G. J., & Jauniaux, E. Oxidative stress. Best Practice & Research. *Clinical Obstetrics & Gynaecology* **49**, 3–16 (2018) https://doi.org/10.1016/j.bpobgyn.2017.06.003
17	Thyroid	3	Song, Y., & Kopp, P. Thyroid hormone synthesis and secretion. *Endotext. MDText.com*. (2018). https://doi.org/10.1210/endo.2017-00250
18	Bone	3	Alexander, K. A., Chang, M. K., Maylin, E. R., Kohler, T., Müller, R., Wu, A. C. et al. Osteal macrophages promote in vivo intramembranous bone healing in a mouse tibial injury model. *Journal of Bone and Mineral Research* **26(7),** 1517–1532 (2011). https://doi.org/10.1002/jbmr.354

19	Adipose tissue	4	Chavakis T., Alexaki V.I., Ferrante A.W. Jr. Macrophage function in adipose tissue homeostasis and metabolic inflammation. *Nat. Immunol.* **24**, 1689–1702 (2023). https://doi.org/10.1038/s41590-023-01479-0

According to exposure to stress, injury, or antigenic stimulation as well as metabolic buffering required to preserve homeostasis, the total number of macrophages fluctuated significantly among tissues (**Table S1-T1**). The liver and bone marrow had the highest scores (scoring 5), which is in accordance with their functions in haematopoiesis and detoxification. High macrophage density was additionally observed in the spleen, lung, uterus, placenta, and adipose tissue (scoring 3-4). On the other hand, tissues such as the kidney, heart, brain, pancreas, skeletal muscles, and testis exhibited minimal macrophage presence (scoring 1).

This scoring integrates data on macrophage ontogeny, turnover, and tissue-specific distribution as described in the following sources:

Davies, L. C., Jenkins, S. J., Allen, J. E., Taylor, P. R. Tissue-resident macrophages. *Nature Immunology* **14(10),** 986–995 (2013). https://doi.org/10.1038/ni.2705

Ginhoux, F., & Guilliams, M. Tissue-resident macrophage ontogeny and homeostasis. *Immunity* **44(3**), 439–449 (2016). https://doi.org/10.1016/j.immuni.2016.02.024

Hashimoto, D., Chow, A., Noizat, C., et al. Tissue-resident macrophages self-maintain locally throughout adult life with minimal contribution from circulating monocytes. *Immunity* **38(4),** 792–804 (2013). https://doi.org/10.1016/j.immuni.2013.04.004

Table S1-T2. Macrophage Scoring Criteria

Score	Approximate Macrophage Density
1	Minimal resident population
2	Low
3	Moderate
4	High
5	Specialised filtering organ

Supplementary S2

Tissue Basal Metabolic Rate (BMRt)

Tissue Basal Metabolic Rate (BMRt) represents the intrinsic energetic demand of a tissue under resting, homeostatic conditions. It quantifies the baseline rate of ATP consumption, oxygen utilization, and mitochondrial activity required to maintain essential cellular functions such as:

- ion homeostasis
- membrane potential maintenance
- protein turnover
- basal biosynthesis
- organelle quality control
- low-level repair processes

BMRt is an organ-specific metabolic signature, reflecting the structural, functional, and bioenergetic specialization of each tissue.

Physiological Determinants of BMRt

1. Mitochondrial density and architecture

Tissues with high mitochondrial content (heart, neurons, skeletal muscle) exhibit elevated BMRt due to continuous ATP turnover. Conversely, tissues with sparse mitochondria (cartilage, adipose tissue) show low BMRt.

2. Cellular composition

The metabolic rate depends on the dominant cell types:

- excitable cells (neurons, cardiomyocytes) → high BMRt
- epithelial cells with active transport (intestine, kidney) → medium–high BMRt
- quiescent or matrix-rich tissues (cartilage, bone) → low BMRt

3. Functional workload at rest

Even in basal conditions, some organs sustain continuous activity:

- heart: uninterrupted contraction
- brain: synaptic signaling and ion pumping
- liver: metabolic processing and detoxification
- placenta: nutrient exchange and endocrine activity

4. Vascularization and oxygen delivery

High-flow tissues support higher oxidative metabolism. Low-flow tissues (cartilage, adipose) are metabolically constrained.

5. Endocrine regulation

Hormones such as thyroid hormones, insulin, glucocorticoids, and catecholamines modulate tissue-specific metabolic tone.

Why BMRt is a Foundational Bioenergetic Variable

BMRt captures a purely metabolic dimension of tissue physiology that is:

- independent from macrophage density (M)
- independent from oxidative stress sensitivity (OS)
- stable across individuals
- organ-intrinsic and evolutionarily conserved

This makes BMRt an ideal axis for a multidimensional model of tissue vulnerability.

For the purposes of the bioenergetic vulnerability framework, BMRt is expressed as a 5-point ordinal score:

Score	Interpretation
**1**	Very low metabolic demand (avascular or minimally active tissues)
**2**	Low metabolic demand
**3**	Medium metabolic demand
**4**	High metabolic demand
**5**	Very high metabolic demand (continuous ATP turnover)

Conceptual Role of BMRt in the Bioenergetic Triad

Summary

BMRt combines the tissue's basal functional workload, oxygen consumption, ATP turnover, and mitochondrial density. Based on published quantitative indicators of tissue-specific metabolic activity, BMRt was expressed as a 5-point ordinal score (1 = extremely low, 5 = very high) for modelling purposes. BMRt captures the metabolic reserve and vulnerability of each organ and is a distinct bioenergetic axis which is orthogonal to oxidative stress sensitivity (OS) and macrophage content (M). **Table S2-T1** offers the values of the several organs and tissues.

Table S2-T1. Tissue Basal Metabolic Rate (BMRt) Table — 19 Organs/Tissues

Organ / Tissue	BMRt (1–5)	Reference
Liver	4	Rui L. Energy metabolism in the liver. *Compr. Physiol.* **4**:177–197 (2014). https://doi.org/10.1002/cphy.c130024
Spleen	3	Mebius R.E., Kraal G. Structure and function of the spleen. *Nat. Rev. Immunol.* **5**:606–616 (2005). https://doi.org/10.1038/nri1669
Lung	4	Hsia C.C.W. Respiratory function of the alveolar–capillary membrane. *Compr. Physiol.* **7**:1–15 (2017). https://doi.org/10.1002/cphy.c160016
Bone marrow	4	Cawthorn W.P., Scheller E.L., MacDougald O.A. Adipose tissue stem cells meet bone: a new frontier in skeletal biology. *Nat. Rev. Endocrinol.* **10**:163–173 (2014). https://doi.org/10.1038/nrendo.2013.204

Intestinal epithelium	4	DeBerardinis R.J., Chandel N.S. *Fundamentals of cancer metabolism*. *Cell Metab*. **30**:507–526 (2019). https://doi.org/10.1016/j.cmet.2019.08.004
Skin / Epidermis	3	Proksch E., Brandner J.M., Jensen J.M. The skin: an indispensable barrier. *Exp. Dermatol.* **17**:1063–1072 (2008). https://doi.org/10.1111/j.1600-0625.2008.00786.x
Kidney	3	Bhargava P., Schnellmann R.G. Mitochondrial energetics in the kidney. *Nat. Rev. Nephrol*. **13**:629–646 (2017). https://doi.org/10.1038/nrneph.2017.107
Heart	5	Stanley W.C., Recchia F.A., Lopaschuk G.D. Myocardial substrate metabolism in the normal and failing heart. *Physiol. Rev*. **85**:1093–1129 (2005) https://doi.org/10.1152/physrev.00006.2004
Brain	5	Harris J.J., Jolivet R., Attwell D. Synaptic energy use and supply. *Trends Neurosci*. **35**:700–712 (2012). https://doi.org/10.1016/j.tins.2012.08.004
Pancreas	3	Prentki M., Nolan C.J. Islet β cell failure in type 2 diabetes. *J. Clin. Invest*. **116**:1802–1812 (2006). https://doi.org/10.1172/JCI29103
Skeletal muscle	4	Zurlo F., Larson K., Bogardus C., Ravussin E. Skeletal muscle metabolism is a major determinant of resting energy expenditure. *J. Clin. Invest*. **86**:1423–1427 (1990). https://doi.org/10.1172/JCI114857
Testis	3	Amaral A., Lourenço B., Marques M., Ramalho-Santos J. Mitochondria functionality and sperm quality. *Nat. Rev. Urol*. **10**:430–438 (2013). https://doi.org/10.1038/nrurol.2013.107
Uterus	3	Critchley H.O.D., Maybin J.A., Armstrong G.M., Williams A.R.W. Physiology of the endometrium and regulation of menstruation. *Nat. Rev. Endocrinol*. **16**:391–405 (2020). https://doi.org/10.1038/s41574-020-0341-0
Ovaries	3	May-Panloup P., Boucret L., Chao de la Barca J.M., et al. Ovarian ageing: the role of mitochondria in oocytes and follicles. *Hum. Reprod. Update* **22**:725–743(2016). https://doi.org/10.1093/humupd/dmw028
Vascular endothelium	3	De Bock K., Georgiadou M., Carmeliet P. Role of endothelial cell metabolism in vessel sprouting. *Cell Metab*. **18**:634–647 (2013). https://doi.org/10.1016/j.cmet.2013.10.001
Placenta	4	Kolahi K.S., Valent A.M., Thornburg K.L. Cytotrophoblast metabolic flexibility: a key to placental function. *Sci. Rep*. **7**:5572 (2017). https://doi.org/10.1038/s41598-017-05964-8
Thyroid	4	Mullur R., Liu Y.-Y., Brent G.A. Thyroid hormone regulation of metabolism. *Physiol. Rev*. **94**:355–382 (2014). https://doi.org/10.1152/physrev.00030.2013
Bones	2	Riddle R.C., Clemens T.L. Bone cell bioenergetics and skeletal energy metabolism. *Nat. Rev. Endocrinol*. **13**:303–314 (2017). https://doi.org/10.1038/nrendo.2016.186
Adipose tissue	1	Chouchani E.T., Kajimura S. Metabolic adaptation and thermogenesis in adipose tissue. *Nat. Rev. Mol. Cell Biol*. **20**:395–411 (2019). https://doi.org/10.1038/s41580-019-0133-5

Table S2-T2. BMRt Scoring Criteria

Score	Relative Metabolic Demand
1	Very low
2	Low
3	Moderate
4	High
5	Very High

Supplementary S3

Intrinsic Sensitivity and Vulnerability Index (ISVI)

The Intrinsic Sensitivity and Vulnerability Index (ISVI) is a composite, dimensionless parameter developed to quantify the inherent susceptibility of a given organ or tissue to microplastic-induced damage. The index integrates three biologically grounded determinants of vulnerability: (i) tissue bioenergetic demand, (ii) macrophage density, and (iii) cellular turnover dynamics. Together, these variables capture the structural, metabolic, and immunological features that modulate the organ's capacity to buffer, neutralize, or accumulate microplastic particles.

Rationale

Microplastic toxicity is strongly conditioned by organ-specific characteristics. Tissues with high metabolic rates, limited phagocytic surveillance, or slow cellular renewal are expected to exhibit reduced resilience to particle accumulation and oxidative stress. ISVI operationalizes these determinants into a single quantitative metric suitable for comparative analyses across organ systems.

Parameters included

ISVI incorporates the following standardized components:

Basal Cellular Index (BCI): A normalized measure of tissue bioenergetic demand, derived from published organ-specific oxygen consumption and metabolic activity data.

Macrophage Availability (Ma): A semi-quantitative score reflecting the density and functional availability of resident macrophages within the tissue microenvironment.

Turnover Time (Tt): The characteristic cellular renewal time of the organ, expressed in days and normalized to a reference scale to ensure comparability across tissues.

Computation

ISVI is calculated using the following composite formulation: $ISVI = BCI / Ma \cdot Tt$

where higher values indicate intrinsically greater vulnerability. All variables were normalized to a 0–1 scale prior to computation to avoid dimensional bias and ensure cross-organ comparability.

1. Cellular turnover (Tt)

Sender, R., and Milo, R. The distribution of cellular turnover in the human body. *Nature Medicine* **27**, 45–48 (2021). https://doi.org/10.1038/s41591-020-01182-9

2. Tissue macrophage density and heterogeneity (Ma)

Gordon, S., and Plüddemann, A. Tissue macrophages: heterogeneity and functions. *BMC Biology* **15**, 53 (2017). https://doi.org/10.1186/s12915-017-0392-4

3. Organ-specific metabolic demand (BCI)

Wculek, S. K., Dunphy, G., Heras-Murillo, I., Mastrangelo, A., & Sancho, D. Metabolism of tissue macrophages in homeostasis and pathology. *Cellular & Molecular Immunology* **18**, 118–134 (2021). https://doi.org/10.1038/s41423-020-00626-4

In **Table S3-T1** are reported the sumury of the data

Table S3-T1. Components of the Intrinsic Sensitivity and Vulnerability Index (ISVI) Across 19 Organs

Organ	BCI (0–1)	Macrophage Availability (0–1)	Turnover (0–1)	ISVI (1–5)
Brain	0.95	0.10	0.95	**5**
Heart	0.90	0.15	0.90	**5**
Kidney	0.85	0.20	0.80	**3 - 4**
Lung (alveolar epithelium)	0.70	0.25	0.70	**4**
Pancreas	0.75	0.25	0.75	**4**
Testis	0.70	0.20	0.70	**4**
Bone marrow	0.60	0.60	0.40	**4**
Intestinal epithelium	0.55	0.55	0.35	**4**
Liver	0.65	0.70	0.50	**4**
Spleen	0.40	0.80	0.40	**2**
Skin (epidermis)	0.35	0.60	0.30	**2**
Skeletal muscle	0.30	0.50	0.60	**2**
Uterus	0.35	0.50	0.50	**2**
Ovaries	0.35	0.50	0.50	**2**
Vascular endothelium	0.40	0.55	0.55	**2**
Placenta	0.45	0.70	0.60	**2**
Thyroid	0.25	0.60	0.70	**1**
Bones	0.20	0.70	0.80	**1**
Adipose tissue	0.20	0.50	0.80	**1**

All variables were normalized to a 0–1 scale to ensure cross-organ comparability.

BCI reflects organ-specific metabolic demand; Macrophage availability (Ma) represents the density and functional accessibility of resident phagocytes; Turnover represents the relative renewal rate of the dominant cell population; ISVI is a categorical score (1–5) derived from the combined biological profile of each organ.

Computation

ISVI is calculated using the following composite formulation: **ISVI=BCI/Ma·Tt**

where higher values indicate intrinsically greater vulnerability. All variables were normalized to a 0–1 scale prior to computation to avoid dimensional bias and ensure cross-organ comparability.

Application in the study

ISVI was computed for each organ included in the analysis and used as a fixed biological descriptor in downstream modeling. The index served to stratify organs according to their intrinsic susceptibility

and to interpret observed differences in microplastic accumulation, clearance kinetics, and oxidative burden. ISVI values were not fitted to experimental data but derived exclusively from literature-based physiological parameters. In **Table S3-T2**, are reported the ISVI values and relative reference

Table S3-T2 Values of ISVI (total) and references

**Brain**	5	Herculano-Houzel, S. The human brain in numbers: A linearly scaled-up primate brain. *Frontiers in Human Neuroscience* **5,** 35 (2011) https://doi.org/10.3389/fnhum.2011.00035
**Heart**	5	Laflamme, M. A., & Murry, C. E. Heart regeneration. *Nature* **473**, (7347), 326–335 (2011) https://doi.org/10.1038/nature10147
**Kidney**	4	Humphreys, B. D., Valerius, M. T., Kobayashi, A., Mugford, J. W., Soeung, S., Duffield, J. S. et al. Intrinsic epithelial cells repair the kidney after injury. *Cell Stem Cell* **2(3)**, 284–291 (2008). https://doi.org/10.1016/j.stem.2008.04.004
**Lung (alveolar epithelium)**	4	Barkauskas, C. E., Cronce, M. J., Rackley, C. R., Bowie, E. J., Keene, D. R., Stripp, B. R.,et al. Type 2 alveolar cells are stem cells in adult lung. *Nature* **507**(7491), 190–194 (2013). https://doi.org/10.1038/nature12746
**Pancreas**	4	Kopp, J. L., Dubois, C. L., Schaffer, A. E., Hao, E., Shih, H. P., Seymour, P. A., et al. Sox9+ ductal cells are multipotent progenitors throughout development but do not produce new endocrine cells in the normal or injured adult pancreas. *Developmental Cell* **18(6),** 759–769 (2011). https://doi.org/10.1016/j.devcel.2011.10.012
**Testis**	4	de Kretser, D. M., & Kerr, J. B. The cytology of the testis. In E. Knobil & J. D. Neill (Eds.), *The Physiology of Reproduction* (pp. 1177–1290) (1994). Academic Press. https://doi.org/10.1016/B978-0-12-515400-0.50010-4
**Bone marrow**	3	Morrison, S. J., & Scadden, D. T. The bone marrow niche for haematopoietic stem cells. *Nature* **505**(7483), 327–334 (2014). https://doi.org/10.1038/nature12984
**Intestinal epithelium**	3	Barker, N. Adult intestinal stem cells: Critical drivers of epithelial homeostasis and regeneration. *Nature Reviews Molecular Cell Biology* **15(1)**, 19–33 (2014). https://doi.org/10.1038/nrm3766
**Liver**	3	Michalopoulos, G. K. Liver regeneration. *Nature Reviews Gastroenterology & Hepatology* **14(1**), 40–55 (2017). https://doi.org/10.1038/nrgastro.2017.38
**Spleen**	2	Mebius, R. E., & Kraal, G. Structure and function of the spleen. *Nature Reviews Immunology* **5(8)**, 606–616 (2005). https://doi.org/10.1038/nri1669
**Skin (epidermis)**	2	Blanpain, C., & Fuchs, E. Epidermal stem cells of the skin. *Cell* **137(2),** 314–329 (2009). https://doi.org/10.1016/j.cell.2009.07.031
**Skeletal muscle**	2	Yin, H., Price, F., & Rudnicki, M. A. Satellite cells and the muscle stem cell niche. *Physiological Reviews,* **93(1),** 23–67 (2013). https://doi.org/10.1152/physrev.00043.2012
**Uterus**	2	Gargett, C. E. Uterine stem cells: What is the evidence? *Reproductive Sciences* **14(6)**, 596–603 (2007). https://doi.org/10.1177/1933719107306897
**Ovaries**	2	Edson, M. A., Nagaraja, A. K., & Matzuk, M. M. The mammalian ovary from genesis to revelation. *Endocrine Reviews* **30(6)**, 624–712 (2009). https://doi.org/10.1210/er.2009-0012
**Vascular endothelium**	2	Aird, W. C.. Phenotypic heterogeneity of the endothelium: I. Structure, function, and mechanisms. *Circulation Research* **100(2**), 158–173 (2007). https://doi.org/10.1161/01.RES.0000255691.76142.4a
**Placenta**	2	Burton, G. J., & Fowden, A. L. The placenta: A multifaceted, transient organ. *Journal of Physiology,* **594(23),** 5647–5672 (2015). https://doi.org/10.1113/JP271057
**Thyroid**	1	Dumont, J. E., Lamy, F., Roger, P., & Maenhaut, C. Physiological and pathological regulation of thyroid cell proliferation and differentiation. *Physiological Reviews* **72(3**), 667–697 (1992). https://doi.org/10.1152/physrev.1992.72.3.667
**Bones**	1	Florencio-Silva, R., Sasso, G. R. S., Sasso-Cerri, E., Simões, M. J., & Cerri, P. S. Biology of bone tissue: Structure, function, and factors that influence bone cells. *Journal of Bone Metabolism* **22(2)**, 85–98 (2015). https://doi.org/10.11005/jbm.2015.22.2.85
**Adipose tissue**	1	Rosen, E. D., & Spiegelman, B. M. What we talk about when we talk about fat. *Cell* **156(1–2),** 20–44 (2014). https://doi.org/10.1016/j.cell.2014.02.004

Macrophage content was used in two distinct analytical contexts. For descriptive organ-level scoring, it was expressed on a 1–5 scale to capture the biological magnitude of macrophage infiltration. For the ISVI algorithm, the same variable was normalized to a 0–1 range to ensure comparability with the other independent variables and to avoid scale-driven dominance within the composite index.

This transformation is monotonic and preserves the biological ordering of the variable. The two representations therefore serve different purposes and do not introduce redundancy or bias.

Normalization Procedure and Category Assignment

Because the physiological variables included in the bioenergetic framework (macrophage abundance, tissue metabolic demand, and cellular turnover) are reported in the literature using heterogeneous units, methodologies, and scales, direct quantitative comparison across organs is not feasible. To enable cross-organ integration while preserving biological ranking, all variables were transformed into standardised dimensionless values and subsequently assigned to ordinal categories.

- **Data Harmonisation**

  For each organ, literature-derived physiological indicators were collected from peer-reviewed sources. Depending on the variable, these included:

  - macrophage density estimates (cells/mm², cells/g tissue, immunohistochemical abundance);
  - oxygen consumption rates;
  - ATP turnover estimates;
  - mitochondrial density;
  - cellular renewal times (days).

  Because these measurements originate from different experimental platforms and cannot be directly compared, a min–max normalization procedure was applied.

For each variable (X), the normalized value (X_n) was calculated according to:

$X_n = (X_i - X_{min}) / (X_{max} - X_{min})$

where:

- X_i = value for the specific organ;
- X_{min} = lowest value observed among the 19 organs;
- X_{max} = highest value observed among the 19 organs.

This transformation generates a dimensionless value ranging from 0 to 1.

For variables in which higher values correspond to lower biological vulnerability (e.g., macrophage abundance), the normalized values were retained without inversion because their protective role is explicitly incorporated into the ISVI denominator.

To facilitate interpretation and maintain consistency with semi-quantitative physiological scoring systems frequently used in pathology and toxicology, normalized values were converted into a five-point ordinal scale.

The following thresholds were applied:

Normalized Value	Category	Score
0.00 – 0.20	Very low	1
0.21 – 0.40	Low	2
0.41 – 0.60	Moderate	3
0.61 – 0.80	High	4
0.81 – 1.00	Very High	5

The use of ordinal categories was chosen for three reasons:

1. Published physiological datasets are heterogeneous and frequently non-comparable across organs.
2. Relative biological ranking is more robust than absolute numerical values when integrating data from different methodologies.
3. The objective of the framework is comparative susceptibility assessment rather than precise physiological quantification.

Consequently, the 1–5 scoring system should be interpreted as a biologically informed ranking tool that preserves relative differences among tissues while avoiding overinterpretation of heterogeneous quantitative datasets.

Normalized variables were used directly in the computation of the Intrinsic Sensitivity and Vulnerability Index (ISVI):

$$\mathrm{ISVI} = \frac{\mathrm{BCI}}{(\mathrm{Ma} \times \mathrm{Tt})}$$

where:

- **BCI** = normalized basal cellular metabolic demand;
- **Tt** = normalized cellular turnover parameter;
- **Ma** = normalized macrophage availability.

The resulting continuous ISVI values were subsequently converted into the same five-category scale (1–5) to facilitate comparison with BMRt and macrophage abundance rankings.

This procedure ensures that all three dimensions of the bioenergetic framework are expressed on a common interpretative scale while preserving their independent physiological meaning.

Suppementary S4

Clinical Contextualisation of the Vulnerability Model (Chicago Cluster)

The constellation of clinical signs and symptoms that have been frequently observed in humans with confirmed exposure to and retention of MNPs is referred to as the "Chicago Cluster." Cardiovascular, neurological, endocrine-metabolic, and immune system signs are included in this cluster, demonstrating a pattern of multisystem involvement rather than discrete organ dysfunction. The Chicago Cluster is used as an exploratory clinical comparator to assess whether the organ, systems identified by the bioenergetic framework overlap with clinical domains that have been reported in individuals with documented MNP exposure. It should not be considered an independent validation of the proposed model. The Chicago Cluster is presented in this study as a hypothesis-generating clinical framework and should not be interpreted as a formally recognised disease entity, validated diagnostic syndrome, or consensus-based clinical classification. At present, the proposed cluster has not undergone independent external validation by other research groups, professional societies, or regulatory bodies. Its inclusion in the present work is intended solely to provide a descriptive clinical context for the interpretation of tissue-specific vulnerability patterns identified by the bioenergetic model. Consequently, associations between the Chicago Cluster and micro- or nanoplastic exposure should be regarded as exploratory and hypothesis-generating rather than demonstrative of causality. The 26 signs and symptoms comprising the corresponding Chicago Cluster and relative attribution number are presented in **Table S4-T1.**

Table S4-T1. Chicago Cluster signs and symptoms

Sygn/ Symptom	Description
1	Bloating, irregular bowel habit, dysbiosis
2	Abdominal pain
3	Dyspeptic symptoms (e.g, nausea, vomot, loss of appetite
4	Blood in feces
5	Chronic non allergic dermatitis
6	Allergies
7	Cough, dyspnea
8	Menstrual changes
9	Chamges in sexuality
10	Behavioral changes
11	Cognitive changes
12	Persisten fatigue
13	Persisten headache
14	Visual disturbances
15	Sensorial diseases (e.g. anosmia, dysgeusia, balace disorders)
16	Hypertension
17	Unexpecte fever
18	Slightly elevated CRP. ESR, cytokines
19	Lymphedema, venous insufficiency, recurrent swelling
20	Any ultrasound alteration (i.e., arterial, venous, intestinal. Kidney)
21	Pancreatic enzyme alteration
22	Liver enzyme alteration
23	Dyslipidemia
24	Creatinine alteration

25	Blood disorders (i.e., anemia, coagulation)
26	Fasting blood glucose increase
	Environmental exposure
27	1 Lives in urban area with high poluution/traffic
28	Regularly driks bottled water
29	Diet reachs small fish seas food
30	Works with plastics, textiles, or industrial setting

The Bayesian classification method was developed as an exploratory statistical tool within the proposed framework and should be interpreted as hypothesis-generating pending independent validation in external cohorts. We established that the presence of at least eight features—six clinical manifestations from the Chicago Cluster plus two exposure indicators—corresponds to a posterior probability of ≥95% for microplastic involvement by combining symptom frequency with environmental exposure variables. The low prior probability of verified MNP-related illness and the high discriminative value of the selected symptoms are represented in this threshold, that is not arbitrary. Consequently, the Bayesian structure minimises the risk of overattribution while capturing clinically significant patterns by providing a statistically validated, repeatable criterion for identifying diseases likely affected by MNP contamination.

In **Table S4-T2** are reported the details of the classification of organ and tissue System by Cluster and Pathophysiological Attribution

Table S4-T2. Functional Organ/Tissue Clusters and Corresponding Clinical Domain

ORGAN /T ISSUE	CLUSTER	ATTRIBUTION
Brain – neurons- glia	**Ce S**	Includes all central nervous system disorders: cognitive, behavioral, neurodegenerative, motor, and sensory (eyes, ears, vestibular).
Hematological/Bone marrow	**H**	Any alteration in blood cell counts, hematopoiesis, immune cell production, or coagulation pathways.
Placenta	**G E**	Conditions arising during pregnancy and delivery, including placental dysfunction, insufficiency, inflammation, or vascular complications.
Intestinal epithelium	**I**	All gastrointestinal disorders: altered bowel habits, dysbiosis, abdominal pain, bloating, malabsorption, inflammatory conditions.
Uterus	**G E**	Menstrual disorders, endometrial dysfunction, uterine inflammation, fibroids, and any structural or functional uterine condition.
Vascular endothelium	**Ca**	Hypertension, atherosclerosis, endothelial dysfunction, venous insufficiency, lymphatic disorders, microvascular disease.
Spleen	**H**	Disorders affecting blood filtration, immune cell turnover, splenic enlargement, or altered blood crasis.
Heart (myocardium)	**Ca**	All cardiac diseases: ischemic, structural, inflammatory, arrhythmic, or metabolic cardiomyopathies.
Liver	**L**	Any hepatic condition: metabolic, inflammatory, cholestatic, fibrotic, or toxic liver disease.
Kidney	**U**	Renal and urological disorders: filtration impairment, tubular dysfunction, nephritis, stones, urinary tract pathology.
Lung (alveolar epithelium)	**R**	All respiratory diseases: obstructive, restrictive, infectious, inflammatory, or vascular pulmonary conditions.
Ovaries	**G E**	Ovarian disorders of reproductive or endocrine origin: follicular dysfunction, cysts, ovulatory disorders, hormonal imbalance.
Testis	**A E**	Testicular diseases including endocrine dysfunction, spermatogenic impairment, inflammation, or structural abnormalities.
Pancreas – endocrine+exocrine	**I E**	Pancreatic diseases affecting digestion or endocrine regulation: pancreatitis, enzyme deficiency, diabetes, metabolic dysfunction.
Skin / Epidermis	**Co S**	All dermatological conditions: inflammatory, infectious, autoimmune, structural, or barrier-related.
Thyroid	**T**	Thyroid dysfunction: hypo/hyperthyroidism, autoimmune thyroiditis, nodules, structural abnormalities.

Skeletal muscle	**Co S**	Muscular diseases and sensory alterations: myopathies, fatigue syndromes, neuromuscular interface disorders.
Bones	**Co**	Bone diseases: metabolic, structural, degenerative, inflammatory, or traumatic.
Adipose tissue	**L E**	Lipodystrophy
Oncology	**O**	Any type of cancer, regardless of tissue of origin.

a = Oncology means that any cancer related to the 19 tissue/organs can be caused by MNPs contaminants

To prevent arbitrary mapping, symptoms in Table S4-T3 were linked to specific organs using strict physiological criteria:

1. **Systemic Inflammatory Signals (i.e., elevated hsCRP/ESR):** Assigned exclusively to tissues driving systemic acute-phase responses (Liver) or those containing major active immune filtering networks (Spleen, Bone Marrow, Lung).
2. **Metabolic and Perfusion Symptoms (i.e., Fatigue, Headache):** Mapped to tissues with high resting metabolic workloads (BMRt=5) and minimal endogenous energy reserves, where localized microvascular dysfunction or ATP depletion quickly triggers clinical signs (Brain, Heart).

The list of the 41 diseases (22 acute and 19 chronic) which determine the 26 signs / symptoms of the Chicago Cluster are listed in **Table S4-T3** and the most common sign/simptoms in **Table S4-T4**.

Table S4-T3. Unified Table — Diseases and Symptoms considerd as part of MIC-Syn

Condition/disease	Symptoms
Autoimmune thyroiditis	Fatigue, menstrual irregularities, cognitive impairment, dyslipidemia, headache, ultrasound abnormalities, joint pain, weight changes, depression
Type 2 diabetes mellitus	High blood glucose, dyslipidemia, hypertension, fatigue, elevated liver enzymes, elevated creatinine, obesity, insulin resistance, polyuria/polydipsia, chronic complications (retinopathy, nephropathy, neuropathy)
Metabolic syndrome	Hypertension, dyslipidemia, high blood glucose, elevated liver enzymes, fatigue, abdominal bloating
Acute food poisoning	Nausea, vomiting, diarrhea, fever, abdominal pain, dysbiosis
Gastroenteritis (viral/bacterial)	Nausea, vomiting, abdominal pain, diarrhea, fever, dysbiosis, elevated CRP, hematemesis/melena (in erosive forms)
Acute appendicitis	Abdominal pain, nausea, vomiting, fever, elevated CRP, ultrasound abnormalities
Inflammatory bowel disease (Crohn’s, ulcerative colitis)	Abdominal pain, blood in stool, dyspepsia, fatigue, elevated CRP, bowel habit changes, fever, anemia
Acute diverticulitis	Left lower quadrant abdominal pain, fever, blood in stool, elevated CRP, ultrasound abnormalities, leukocytosis, bowel habit changes
Ischemic colitis	Abdominal pain, blood in stool, nausea, vomiting, elevated CRP, ultrasound abnormalities
Acute pancreatitis	Abdominal pain (severe epigastric radiating to back), nausea, vomiting, fever, elevated amylase/lipase
Acute cholecystitis	Abdominal pain, fever, nausea, vomiting, ultrasound abnormalities, elevated CRP
Chronic pancreatitis	Recurrent abdominal pain, elevated pancreatic enzymes, dyspepsia, fatigue, ultrasound abnormalities, blood test abnormalities, steatorrhea, secondary diabetes, pancreatic calcifications
Acute viral/toxic hepatitis	Fever, nausea, vomiting, elevated liver enzymes, dyslipidemia, persistent fatigue, jaundice, dark urine
Cholelithiasis (gallstones)	Postprandial biliary colic, right upper quadrant pain, nausea/vomiting
Rheumatoid arthritis (RA)	Fatigue, headache, anemia, elevated CRP, cognitive impairment, sexual dysfunction, menstrual irregularities, symmetric joint pain, morning stiffness >30 min, joint swelling, chronic deformities, rheumatoid nodules
Systemic lupus erythematosus (SLE)	Fatigue, headache, cognitive impairment, dermatitis, anemia, elevated CRP, menstrual irregularities
Scleroderma (systemic sclerosis)	Dermatitis, abdominal pain, fatigue, elevated CRP, ultrasound abnormalities, blood test abnormalities, Raynaud phenomenon, dysphagia, pulmonary fibrosis, pulmonary hypertension
Fibromyalgia	Fatigue, headache, cognitive impairment, abdominal pain, behavioral changes, sensory disturbances, sleep disturbances
Chronic bronchitis (COPD)	Chronic productive cough (>3 months/year for ≥2 years), dyspnea, progressive fatigue, elevated CRP, cognitive impairment, hematologic abnormalities, ultrasound abnormalities, cyanosis, right heart hypertrophy
Sarcoidosis	Dry cough, dyspnea, fatigue, headache, elevated CRP, ultrasound abnormalities, cognitive impairment, skin lesions
Deep vein thrombosis (DVT)	Swelling, pain, ultrasound abnormalities, fever, elevated CRP, venous insufficiency
Acute allergic reaction	Allergies, dyspnea, edema, behavioral changes, nausea, vomiting
COVID-19	Fever, cough, dyspnea, anosmia, ageusia, fatigue, risk of pneumonia/ARDS
Post-COVID syndrome	Fatigue, headache, cognitive impairment, cough, dyspnea, sensory disturbances
Pyelonephritis	Fever, lumbar pain, nausea, vomiting, elevated creatinine, elevated CRP, dysuria, hematuria
Seasonal influenza	Fever, cough, fatigue, headache, muscle pain, nausea, vomiting
Bacterial endocarditis	Fever, fatigue, ultrasound abnormalities, elevated CRP, anemia, behavioral changes

Multiple sclerosis	Cognitive impairment, visual disturbances, fatigue, behavioral changes, paresthesia, motor weakness, ataxia, asthenia, relapsing–remitting course
Parkinson's disease	Cognitive impairment, behavioral changes, fatigue, bowel disturbances, sensory disturbances, altered sexual behavior, resting tremor, rigidity, bradykinesia, postural instability, mask-like facies
Alzheimer's disease	Altered sexual behavior, behavioral changes, sensory disturbances, elevated CRP, progressive memory loss, cognitive decline, disorientation
Migraine	Recurrent unilateral headache, photophobia, nausea, fatigue, cognitive changes, sensory disturbances, visual aura
Meningitis (bacterial/viral)	Fever, headache, photophobia, cognitive impairment, nausea, vomiting, elevated CRP, neck stiffness
Encephalitis (bacterial/viral)	Fever, cognitive impairment, behavioral changes, headache, nausea, vomiting, seizures, focal neurological signs
Chronic fatigue syndrome	Fatigue, cognitive impairment, headache, behavioral changes, sensory disturbances
Major depressive disorder	Cognitive impairment, behavioral changes, fatigue, dyspepsia, headache, sexual dysfunction, persistent sadness, anhedonia, sleep disturbances, suicidal ideation
Acute cystitis (UTI)	Pelvic pain, dysuria, fever, nausea, elevated creatinine, elevated CRP, urinary frequency, urgency, hematuria, cloudy urine
Chronic kidney disease	Elevated creatinine, hypertension, fatigue, ultrasound abnormalities, blood test abnormalities, elevated CRP, pruritus, renal osteodystrophy, uremia
Polycystic ovary syndrome (PCOS)	Menstrual irregularities, altered sexuality, dyslipidemia, high blood glucose, fatigue, elevated liver enzymes, hirsutism, acne, obesity, infertility, polycystic ovaries on ultrasound

The frequency of signS and symptoms in the 41 dieases part of MIC-Syn are listed in **Table S4-T4**.

Table S4-T4. Sygn and Symptoms frequency

Sygn/Symptom	Frequency (n/41)	%Category
Persistent fatigue	26	65.0
Dyspeptic symptoms	23	57.5
CPR increase	22	55.0
Unexpecetd Fever	19	47.5
Headache	18	45.0
Cognitive alteration	17	42.5
Blood disorders	16	40.0
Ultrasound alteration	15	37.5
Abdominal pain	15	37.5
Dysbiosis	13	32.5
Sensorial alteration	10	25.0
Behavioral alteration	8	20.0
Cough/dyspnea	7	17.5
Liver enzyme alteration	5	12.5
Dyslipidemia	5	12.5
Creatinine alteration	5	12.5
Sexuality alteration	4	10.0
Visual alteration	4	10.0
Hypertension	4	10.0
Glucose alteration	4	10.0
Menstrual alteration	3	7.5
Blood in stools	3	7.5
Dermatitis	2	5.0
Pancreas enzyme aleration	2	5.0
Allergies	1	2.5
Vascular alteration	1	2.5

Suppementary S5

The distribution of symptoms indicates a multisystem; the most prevalent symptoms are physical fatigue (symptom 12), discomfort in the abdomen (symptom 1), and higher ER/CRP (symptom 18). The brain is influenced by fatigue, the gastrointestinal tract is mostly affected by abdominal pain, and increased ER/CRP is indicative of persistent low-grade inflammation. The MI-Syn framework encompasses all disorders associated with MNPs. Overall, the bioenergetic framework developed in this study substantially reflects the Chicago Cluster [1].

Integration and harmonization

All three parameters were assigned independently and then harmonized across tissues to ensure internal consistency. The resulting matrix in **Table S4-T5** provides a semi-quantitative, physiologically grounded framework for modeling organ-specific vulnerability to microplastic-induced metabolic, inflammatory, and oxidative stress. The approach is reproducible and can be updated as new quantitative data become available.

Table S4-T5. Final Integrated Bioenergetic sensitivity (Descending Total Score)

Organ / Tissue	Ma	BMRt	ISVI	Total	Chicago Cluster
Bone marrow	4	5	3	12	**H**
Spleen	5	3	3	11	**H**
Lung (alveolar epithelium)	4	3	4	11	**L**
Intestinal epithelium	3	5	3	11	**I**
Liver	4	3	3	10	**L**
Kidney	3	3	4	10	**U**
Pancreas	3	3	4	10	**I E**
Vascular endothelium	3	4	4	10	**Ca**
Placenta	**3**	4	4	10	**G E**
Testis	2	3	5	10	**AE**
Skin (epidermis)	3	4	2	9	**Co S**
Heart	2	2	5	9	**Ca**
Uterus	3	3	3	9	**G E**
Ovaries	2	2	4	8	**G E**
Thyroid	2	3	3	8	**T**
Brain	1	1	5	7	**Ce S**
Adipose tissue	3	2	2	7	**L E**
Skeletal muscle	**2**	2	2	6	**Co S**
Bones	2	2	2	6	**Co**
Oncology					**O**

Ma = macrophages content; BMRt= Basal Mtebolic Rrate; ISVI = Intrinsic Sensitivity Vulmerability Index Legenda: C_e=Cerebral; H = hematological; I = Intestinal; C_a= Cardiovascular; G =Gynecological; O = Oncological; Co=Connective; L= Liver; U = Urological; S = Sensorial; T = Thyroid: E = Endocrine; R =Respiratory.

The difference of > 1 point among the organs can be considered important. Bone marrow ranks highest scoring 12 and share a unique constellation of bioenergetic and structural features that make it the most vulnerable and most metabolically demanding in the entire organism. Most probably the tissue was should be easier to find early markers of the MNP contamination.

Organs/tissue scoring 11 points have High metabolic throughput, high oxygen exposure, high turnover, systemic centrality, and perfusion dependency. Each organ is a hub; Bone marrow: hematopoietic hub; Spleen: immunological hub; Lung: respiratory hub; Intestine: nutritional hub. These tissue also can give indications of early MNP contamination

By contrast, organs scoring 10 or below exhibit intermediate bioenergetic demands, supported by greater structural turnover, partial functional redundancy, or more flexible metabolic strategies.

Bioenergic Panel Index

Suggestion of Bioenergetic Damage Panel (3-marker framework)

The lab analysis that can give early indication of MNP syndrome was considered on the base of the bioenergetics theory. A minimal set of three blood-based variables can capture early bioenergetic distress across the organs ranked in the upper tiers of the Tables S5-T1–S5-T4 matrix (>10), integrating oxidative load, perfusion mismatch, cytolytic stress, and myocardial vulnerability.

1**. Plasma lactate.** It is sensitive indicator of impaired oxidative phosphorylation and oxygen-delivery mismatch, capturing early failure in high-throughput, oxygen-dependent tissues such as myocardium, neurons, liver, and alveolar epithelium. Consistent with the role of plasma lactate as an early indicator of oxygen-delivery mismatch [2]) and impaired oxidative phosphorylation under metabolic stress [3], elevated lactate provides a sensitive, system-level readout of bioenergetic strain across high-throughput organs scoring ≥10 in the matrix.

3. Serum LDH. It reflects cytosolic stress in high-throughput, high-turnover tissues. In the context of organs scoring ≥10, LDH captures a shared vulnerability: these organs operate with intense glycolytic–mitochondrial coupling, rapid metabolite flux, and continuous structural renewal. Even minimal perturbations in perfusion, redox balance, or cellular integrity lead to small but measurable LDH leakage, making it a sensitive integrator of bioenergetic strain, not of lipid metabolism. In essence is a regulator of the lactate–pyruvate shuttle and redox balance. Consistent with the central role of LDH in the lactate–pyruvate shuttle and redox buffering [4, 5], circulating LDH provides a sensitive readout of cytosolic metabolic strain across high-throughput organs scoring ≥10 in the bioenergetic matrix."

4. High-sensitivity CRP (hs-CRP) is a sensitive marker of systemic inflammatory activation [6], which disproportionately affects high-throughput, perfusion-dependent organs and amplifies oxidative and metabolic stress across the ≥10 group.

These variables were selected not only for their mechanistic relevance but also for their statistical independence, ensuring that thy captures three orthogonal dimensions of vulnerability rather than redundant descriptors of the same phenomenon. This independence was confirmed through Spearman correlation analysis across 19 human organs and tissues, demonstrating that M, BMRt, and OS represent distinct and non-overlapping biological domains.

The recommended trinity of plasma lactate, serum LDH, and hsCRP is not a conclusive diagnostic test for microplastic exposure; rather, it is a wide physiological proxy for cellular metabolic strain. To distinguish chronic MNP friction from acute systemic diseases, these indicators must only be assessed in conjunction with comprehensive exposure registries and confirmed temporal persistence because they are shared with acute infectious, oncological, and ischaemic disorders.

The thereshold for MNP to determine the possibility of a MIC-Syn are reported in **Tables S5-T1–S5-T4**.

Table S5-T1. Clinical Pathological Thresholds for MNP

**Biomarker**	**Unit**	**Normal Range**	**Pathological Threshold**	**Clinical Notes**
**Lactate**	mmol/L	0.5–2.0	> 2.0	>2 mmol/L suggests hypoperfusion; >4 mmol/L with acidosis indicates critical illness.
**LDH**	U/L	120–250 (method-dependent)	> 250	Non-specific marker of tissue injury; >250 strongly suggests acute damage.
**hsCRP**	mg/L	< 1	> 3	>3 mg/L is the key threshold for cardiovascular risk stratification.

The bioenergetics diagnostic is summarized in **Table S4-T2**, **Table S4-T3,** and **Table S4-T4**.

Table S5-T2. MIC-Syn Bioenergetic Diagnostic Table

**Component**	**Normalized Index**	**Diagnostic Threshold**	**Interpretation**
Lactate	Ln=L/2.0	Ln>1	Bioenergetic overload / impaired oxidative metabolism
LDH	Dn=D/250	Dn>1	Cytolytic stress / accelerated cellular turnover
hsCRP	Cn=C/3.0	Cn>1	Systemic inflammatory activation

Table S5-T3 MIC-Syn Bioenergetic Risk Classification

**Condition**	**Logical Rule**	**Interpretation**
No MIC-Syn bioenergetic risk	At least one index ≤ 1	One or more axes remain within physiological limits; no integrated bioenergetic disruption
MIC-Syn bioenergetic risk present	Ln>1 AND Dn>1 AND Cn>1	All three axes simultaneously exceed pathological thresholds, indicating coordinated metabolic, cytolytic, and inflammatory activation

Table S5-T4 Optional: Severity Index (only for MIC-Syn–positive subjects)

**Index**	**Formula**	**Meaning**
MIC-Syn Severity Index	IMIC,sev=Ln+Dn+Cn3	Quantifies the magnitude of bioenergetic disruption once the diagnostic criterion is met; not used for diagnosis

Interpretation

The MIC-Syn bioenergetic framework requires the simultaneous activation of three independent biological axes—metabolic overload (lactate), cytolytic stress (LDH), and systemic inflammation (hsCRP). Each biomarker is normalized to its pathological threshold, generating three dimensionless indices (Ln, Dn, Cn).

MIC-Syn bioenergetic risk is diagnosed only when all three normalized indices exceed 1. This strict AND-logic prevents false positives due to isolated abnormalities and ensures that the syndrome is defined by a coordinated, system-level bioenergetic collapse.

Once the diagnostic criterion is satisfied, the MIC-Syn Severity Index (mean of the three normalized values) can be used to quantify the magnitude of disruption, but it plays no role in establishing the diagnosis.

References

[1] Cornelli, U., Casella, C., Belcaro, G., Cesarone, MR., Marucci, S., Rondanelli, M., Recchia, M., Zanoni, G. Definition of emerging microplastic Syndrome based on clinical and epidemiological evidence: a narrative review. *Microplastics* **4**, 93 (2025). httpss//doi.org/10.3390/microplastics4040093

[2] Bakker, J., and Jansen, T. C. Don't take vitals, take a lactate. *Intensive Care Medicine* **33(11)**, 1863–1865, (2007). https://doi.org/10.1007/s00134-007-0680-6

[3] Kraut, J. A., & Madias, N. E. Lactic acidosis. *New England Journal of Medicine* **11**;371(24):2309-2319 (2014). https//doi.org/10.156/NEJMra1309483

[4] Brooks, G. A. The science and translation of lactate shuttle theory. *Cell Metabolism* **27(4)**, 757–785 (2018). https://doi.org/10.1016/j.cmet.2018.03.008

[5] San-Millán, I., & Brooks, G. A. Reexamining cancer metabolism: Lactate production for carcinogenesis could be the purpose of the Warburg effect. *Carcinogenesis* **38(2)**, 119–133 (2017). https//doi.org/10.1093/carcin/bgw127

[6] Ridker, P. M. High-sensitivity C-reactive protein: Potential adjunct for global risk assessment in the primary prevention of cardiovascular disease. *Circulation* **107(3),** 363–369 (2003) https://doi.org/10.1161/01.CIR.0000053730.47739.3C

Supplementary S6

BIOENERGETIC SPECIFIC ORGANS DETAILS

Bioenergetic implication consists of: Macrophases content (Ma); Basal Metabolic Rate (BMRt) and Intrinsic Sensitivity and Vulnerability Index (ISVI)

LUNG — Cell Turnover, Sources, Bioenergetic Implication

Cell type	Turnover	Typical source	Bioenergetic implication
Type I pneumocytes	~1 year (derived from type II)	^{14}C studies	Slow renewal of alveolar surface → long-term sequestration
Type II pneumocytes	~8 months to >1 year	^{14}C retrospective birth-dating studies in humans	Long-lived epithelial cells → persistent intracellular retention of particles
Alveolar macrophages	~80 days to several months; some subsets persist years	Fate-mapping and human macrophage kinetics	Professional phagocytes with slow turnover → major reservoir for inhaled microplastics

Reference

Type II Type I pneumocytes

Spalding, K. L., Bhardwaj, R. D., Buchholz, B. A., Druid, H., Frisén, J. Retrospective birth dating of cells in humans. *Cell* **122(1)**, 133–143 (2005). https://doi.org/10.1016/j.cell.2005.04.028

Alveolar macrophages: not considered in the turnover of other tissues

Hashimoto, D., Chow, A., Noizat, C., et al. Tissue-resident macrophages self-maintain locally throughout adult life with minimal contribution from circulating monocytes. *Immunity* **38(4)**, 792–804 (2013) https://doi.org/10.1016/j.immuni.2013.04.004

Yona, S., Kim, K. W., Wolf, Y., et al. Fate mapping reveals origins and dynamics of monocytes and tissue macrophages under homeostasis. *Immunity* **38(1)**, 79–91(2013) https://doi.org/10.1016/j.immuni.2012.12.001

Hume, D. A. The many alternative faces of macrophage activation. *Frontiers in Immunology* **6**, 370 (2015). https://doi.org/10.3389/fimmu.2015.00370

Epelman, S., Lavine, K. J., Randolph, G. J. Tissue-resident macrophages and their origins. *Nature Reviews Immunology* **14(12)**, 731–744 (2014) https://doi.org/10.1038/nri3713

LIVER — Cell Turnover, Sources, Bioenergetic Implication

Cell type	Turnover	Typical source	Bioenergetic implication
Hepatocytes	200–300 days	Human ^14C birth-dating studies	Slow parenchymal turnover → progressive intracellular accumulation
Kupffer cells	Months to years	Fate-mapping and macrophage renewal studies	Long-lived phagocytes → strong hepatic reservoir for microplastics
Hepatic stellate cells	Years	ECM turnover studies	Very slow renewal → long-term retention of trapped particles

References

Horvatits, T., Tamminga, M., Liu, B., Sebode, M., Carambia, A., Fischer, L., et al. Microplastics detected in cirrhotic liver tissue. *eBioMedicine* **82**, 104147 (2022) https://doi.org/10.1016/j.ebiom.2022.104147

Beyzaei, Z. B., Geramizadeh, B. G., Bagheri, Z. B., Karimzadeh, S. K., & Weiskirchen, R. Microplastics in focus: A silent disruptor of liver health – A systematic review. *Frontiers in Pharmacology,* **16** (2025). https://doi.org/10.3389/fphar.2025.1543210

KIDNEY — Cell Turnover, Sources, Bioenergetic Implication

Cell type	Turnover	Typical source	Bioenergetic implication
Proximal tubular epithelial cells	~6–10 months (slow renewal)	Human ^14C birth-dating and renal epithelial kinetics	Slow turnover → progressive intracellular accumulation of filtered microplastics
Podocytes	Years; extremely limited regeneration	Human glomerular cell turnover studies	Very long-lived cells → particles remain for years once internalized
Mesangial cells	Months–years	ECM turnover and glomerular remodeling studies	Slow renewal → stable retention of trapped particles in the mesangium
Interstitial macrophages	Months–years	Fate-mapping and renal macrophage renewal studies	Long-lived phagocytes → strong renal reservoir for microplastics
Endothelial cells (glomerular)	~100–150 days	Endothelial turnover studies	Intermediate turnover → partial but persistent accumulation

Bioenergetic interpretation

The kidney combines slow epithelial turnover, extremely long-lived podocytes, and persistent macrophage populations. → This creates a medium–high reservoir, especially in the proximal tubule, mesangium, and interstitial macrophages, fully consistent with autopsy findings of renal microplastic accumulation.

References

Bergmann, M., Mützel, S., Primpke, S., Tekman, M. B., Trachsel, J., & Gerdts, G. White and wonderful? Microplastics prevail in snow from the Alps to the Arctic. *Science* **363,** (6432), 1100–1102 (2019). https://doi.org/10.1126/science.aav6839

Dzierżyński, E., Blicharz-Grabias, E., Komaniecka, I., Panek, R., Forma, A., Gawlik, P. J.et al. Post-mortem evidence of microplastic bioaccumulation in human organs: Insights from advanced imaging and spectroscopic analysis. *Archives of Toxicology* **99**, 4051–4066 (2025) https://doi.org/10.1007/s00204-025-03785-3

HEART — Cell Turnover, Sources, Bioenergetic Implication

Cell type	Turnover	Typical source	Bioenergetic implication
Cardiomyocytes	Years to decades (≈1%/year in youth; 0.3%/year in elderly)	Human ^14C birth-dating	Extremely slow turnover → long-term retention of microplastics once internalized
Cardiac fibroblasts	Months–years	ECM turnover studies	Slow renewal → persistent interstitial accumulation
Cardiac macrophages	Months–years	Fate-mapping studies	Long-lived phagocytes → stable reservoir for particles
Endothelial cells	~100 days	Endothelial kinetics	Intermediate turnover → partial clearance but persistent load

References

Bergmann, O., Bhardwaj, R. D., Bernard, S., Zdunek, S., Barnabé-Heider, F., Walsh, S., et al. Evidence for cardiomyocyte renewal in humans. *Science* **324**, 98–102 (2009). https://doi.org/10.1126/science.1164680

Dzierżyński, E., et al. Post-mortem evidence of microplastic bioaccumulation in human organs. *Archives of Toxicology* **99**, 4051–4066 (2025). https://doi.org/10.1007/s00204-025-03785-3

BRAIN — Cell Turnover, Sources, Bioenergetic Implication

Cell type	Turnover	Typical source	Bioenergetic implication
Neurons	Decades to lifelong	Human ^14C birth-dating	Essentially no turnover → particles persist for life
Microglia	~4–15 months	Microglial kinetics studies	Long-lived phagocytes → strong reservoir for microplastics
Astrocytes	Years	Glial turnover studies	Slow renewal → long-term retention
Endothelial cells (BBB)	~100 days	Endothelial turnover	Intermediate turnover → partial but persistent accumulation

References

Neurons *lifelong*

Spalding, K. L., Bergmann, O., Alkass, K., Bernard, S., Salehpour, M., Huttner, H. B.,et al. Dynamics of hippocampal neurogenesis in adult humans. *Cell* **153**, 1219–1227 (2013). https://doi.org/10.1016/j.cell.2013.05.002

Microglia *~4–15months*

Reu, P., Khosravi, A., Bernard, S., Mold, J. E., Salehpour, M., Alkass, K., Perl, S., Tisdale, J., Possnert, G., Druid, H., & Frisén, J. The lifespan and turnover of microglia in the human brain. *Cell Reports* **20(4)**, 779–784 (2017). https://doi.org/10.1016/j.celrep.2017.07.004

Astrocytes -*Years*

Bergmann, O., Liebl, J., Bernard, S., Alkass, K., Yeung, M. S. Y., Steier, P., Kutschera, W., Johnson, L., Landén, M., Druid, H., & Frisén, J. The age of olfactory bulb neurons in humans. *Neuro* **74(4)**, 634–639 (2012). https://doi.org/10.1016/j.neuron.2012.03.030

Cerebral endothelium (BBB) ***~100 days***

Daneman, R., & Prat, A. The blood–brain barrier. *Cold Spring Harbor Perspectives in Biology*, **7(1),** a020412 (2015). https://doi.org/10.1101/cshperspect.a020412

PLACENTA — Cell Turnover, Sources, Bioenergetic Implication

Cell type	Turnover	Typical source	Bioenergetic implication
Syncytiotrophoblast	~3–7 days	Placental renewal studies	Rapid turnover → lower long-term retention but high transient exposure
Cytotrophoblasts	~48–72 hours	Trophoblast kinetics	Rapid renewal → particles transferred to fetus or maternal circulation
Hofbauer cells (placental macrophages)	Months	Placental immunology	Long-lived phagocytes → local reservoir for microplastics
Endothelial cells	~100 days	Endothelial turnover	Intermediate retention

References

Ragusa, A., Svelato, A., Santacroce, C., Catalano, P., Notarstefano, V., Carnevali, et, al. Plasticenta: First evidence of microplastics in human placenta. *Environment International* **146**, 106274 (2021) https://doi.org/10.1016/j.envint.2020.106274

Macrophages not considered

GASTROINTESTINAL TRACT — Cell Turnover, Sources, Bioenergetic Implication

Cell type	Turnover	Typical source	Bioenergetic implication
Enterocytes	3–5 days	Intestinal epithelial kinetics	Very fast turnover → low retention but continuous exposure
Goblet cells	~5 days	Mucosal turnover	Rapid renewal → transient retention
Paneth cells	~6–8 weeks	Crypt cell studies	Moderate retention → potential microplastic reservoir
Intestinal macrophages	Months	Gut immunology	Long-lived → persistent accumulation in lamina propria

References

Enterocyites - Goblet cells - ***3-5 days***

Darwich, A. S., Aslam, U., Ashcroft, D. M., & Rostami-Hodjegan, A. Meta-analysis of the turnover of intestinal epithelia in preclinical animal species and humans. *Drug Metabolism and Disposition* **42(12)**, 2016–2022 (2014). https://doi.org/10.1124/dmd.114.059188

Yu, L.E., Yang, W.C., & Liang, Y.C. Crosstalk within the intestinal epithelium: Aspects of intestinal absorption, homeostasis, and immunity. *Biomedicines* **12(12),** 2771 (2024). https://doi.org/10.3390/biomedicines12122771

Paneth cells ***6–8 weeks***

Quintero, M., Keeley, T. M., Colacino, J., Tapaswi, A., & Samuelson, L. C. Intestinal stem cells remodel in response to acute Notch inhibition and Paneth cell loss. *Physiology* **39(S1)** (2024). https://doi.org/10.1152/physiol.2024.39.S1.1196

Goga, A., Yagabasan, B., Herrmanns, K., et al. miR-802 regulates Paneth cell function and enterocyte differentiation in the mouse small intestine. *Nature Communications* **12**, 2950 (2021). https://doi.org/10.1038/s41467-021-23298-3

Macrofages ***-months***

Epelman, S., Lavine, K. J., & Randolph, G. J.). Tissue-resident macrophages and their origins. *Nature Reviews Immunology* **14(12)**, 731–744 (2014 https://doi.org/10.1038/nri3713

Hume, D. A. *The many alternative faces of macrophage activation. Frontiers in Immunology* **6**, 370 (2015). https://doi.org/10.3389/fimmu.2015.00370

ADIPOSE TISSUE — Cell Turnover, Sources, Bioenergetic Implication

Cell type	Turnover	Typical source	Bioenergetic implication
Adipocytes	~8–10 years (≈10% renewed per year)	Human ^14C birth-dating	Extremely slow turnover → long-term storage of lipophilic microplastics
Stromal vascular cells	Months–years	Adipose biology studies	Slow renewal → persistent interstitial accumulation
Adipose macrophages	Months–years	Fate-mapping	Long-lived phagocytes → strong reservoir for microplastics

References

Spalding, K. L., Arner, E., Westermark, P. O., Bernard, S., Buchholz, B. A., Bergmann, et.al Dynamics of fat cell turnover in humans. *Nature* **453**, 783–787 (2008). https://doi.org/10.1038/nature06902

Dzierżyński, E., et al. Post-mortem evidence of microplastic bioaccumulation in human organs. *Archives of Toxicology* **99**, 4051–4066 (2025). https://doi.org/10.1007/s00204-025-03785-3

OVARY — Cell Turnover, Sources, Bioenergetic Implication

Cell type	Turnover	Typical source	Bioenergetic implication
Ovarian surface epithelium (OSE)	~7–10 days	OSE renewal studies	Rapid turnover → low long-term retention, but continuous exposure
Granulosa cells	Days–weeks	Folliculogenesis kinetics	Rapid renewal → transient retention
Theca cells	Weeks	Follicular remodeling studies	Moderate retention
Ovarian macrophages	Months	Ovarian immunobiology	Long-lived phagocytes → local reservoir for microplastics
Stromal fibroblasts	Months–years	Ovarian stromal turnover	Slow turnover → persistent interstitial accumulation

References

Ovarian surface epithelium (OSE) — *days*

Auersperg, N., Wong, A. S. T., Choi, K.-C., Kang, S. K., & Leung, P. C. K. Ovarian surface epithelium: Biology, endocrinology, and pathology. *Endocrine Reviews* **22(2)**, 255–288 (2001). https://doi.org/10.1210/edrv.22.2.0422

Granulosa cells — *weeks*

Schütz, L. F., & Batalha, I. M. Granulosa cells: Central regulators of female fertility. *Endocrines* **5(4)**, 547–565 (2024). https://doi.org/10.3390/endocrines5040040

Theca cells — *weeks*

Richards, J. S., & Pangas, S. A. The ovary: Basic biology and clinical implications. *Journal of Clinical Investigation* **120(4)**, 963–972 (2010). https://doi.org/10.1172/JCI41350

Ovarian macrophages — months not considered

Tang, M., Zhao, M., & Shi, Y. New insight into the role of macrophages in ovarian function and ovarian aging. *Frontiers in Endocrinology* **14**, 1282658 (2023). https://doi.org/10.3389/fendo.2023.1282658

Stromal fibroblasts — *Years*

Weicheng, T., Wang, K., Feng, Y., et al. Exploration of the mechanism and therapy of ovarian aging by targeting cellular senescence. *Life Medicine* **4(1)**, lnaf004 (2025). https://doi.org/10.1093/lifemedi/lnaf004

THYROID — Cell Turnover, Sources, Bioenergetic Implication

Cell type	Turnover	Typical source	Bioenergetic implication
Thyrocytes (follicular cells)	~80–120 days	Thyroid epithelial kinetics	Slow turnover → progressive intracellular accumulation
Parafollicular C-cells	Months–years	Endocrine cell turnover studies	Long-lived → persistent retention
Thyroid macrophages	Months–years	Thyroid immunology	Strong reservoir for microplastics
Stromal fibroblasts	Months–years	ECM turnover	Long-term interstitial accumulation

References

Dumont, J. E., Lamy, F., Roger, P., & Maenhaut, C. Physiology of the thyroid gland. *Physiological Reviews* **72**, 667–697 (1992). https://doi.org/10.1152/physrev.1992.72.3.667

Dzierżyński, E., et al. Post-mortem evidence of microplastic bioaccumulation in human organs. *Archives of Toxicology* **99**, 4051–4066 (2025). https://doi.org/10.1007/s00204-025-03785-3

CAROTID ARTERY — Cell Turnover, Sources, Bioenergetic Implication

Cell type	Turnover	Typical source	Bioenergetic implication
Endothelial cells	~100 days	Vascular endothelial turnover	Intermediate turnover → partial retention
Vascular smooth muscle cells (VSMCs)	Years	Arterial wall kinetics	Very slow turnover → long-term retention of particles
Adventitial fibroblasts	Months–years	ECM turnover	Persistent interstitial accumulation
Arterial macrophages	Months–years	Atherosclerosis immunobiology	Strong reservoir, especially in plaques

References

Clarke, M. C. H., Figg, N., Maguire, J. J., Davenport, A. P., Goddard, M., Littlewood, T. D., & Bennett, M. R. Apoptosis of vascular smooth muscle cells induces features of plaque vulnerability in atherosclerosis. *Nature Medicine* **12**, 1075–1080 (2006). https://doi.org/10.1038/nm1459

Dzierżyński, E., et al. Post-mortem evidence of microplastic bioaccumulation in human organs. *Archives of Toxicology* **99**, 4051–4066 (2025). https://doi.org/10.1007/s00204-025-03785-3

AORTA — Cell Turnover, Sources, Bioenergetic Implication

Cell type	Turnover	Typical source	Bioenergetic implication
Aortic endothelial cells	~100 days	Endothelial turnover studies	Intermediate retention
Aortic smooth muscle cells	Years to decades	Human arterial wall kinetics	Extremely slow turnover → major long-term reservoir
Adventitial fibroblasts	Months–years	ECM remodeling	Persistent interstitial accumulation
Aortic macrophages	Months–years	Vascular immunology	Strong reservoir, especially in inflamed or aged aortas

References

Bennett, M. R., Sinha, S., & Owens, G. K. Vascular smooth muscle cells in atherosclerosis. *Circulation Research* **118**, 692–702 (2016). https://doi.org/10.1161/CIRCRESAHA.115.306361

Dzierżyński, E., et al. Post-mortem evidence of microplastic bioaccumulation in human organs**.** *Archives of Toxicology* **99**, 4051–4066 (2025). https://doi.org/10.1007/s00204-025-03785-3

SKIN- Cell Turnover – Source – Bioenergetic Implication

Organ	**Turnover**	**Typical Source**	**Bioenergetic Implication**
Epidermal	High turnover (epidermis renews every ~28 days; accelerated under stress)	Keratinocytes, fibroblasts, melanocytes; lipid matrix; microvascular and immune networks	High mitochondrial demand for differentiation, collagen synthesis, barrier maintenance, redox signaling. Sensitive to UV, pollutants, thermal stress. Early sentinel of systemic oxidative imbalance.
Derma fibroblsts	Very low	Months years	Slow turnover → prolonged intracellular retention of xenobiotics and persistent oxidative burden. Acts as a long-term reservoir for microplastics and a chronic amplifier of local inflammatory signaling.

References

Epidermal ***28-42 days***

Maeda, K.**).** New method of measurement of epidermal turnover in humans. *Cosmetics* **4(4)**, 47 (2017). https://doi.org/10.3390/cosmetics4040047

Dermal fibroblasts ***–months-years***

Quan, T.. *Human skin aging and the anti-aging properties of retinol.* Biomolecules **13**, 1614. https://doi.org/10.3390/biom13111614 (doi.org in Bing) Naharro-Rodriguez, J., et al. Decoding skin aging: A review of mechanisms, markers, and modern therapies. *Cosmetics* **12(4**), 144 (2023). https://doi.org/10.3390/cosmetics12040144

SPLEEN — Cell Turnover, Sources, Bioenergetic Implications

Cell type (Spleen)	Turnover	Typical source	Bioenergetic implication
**Splenic dendritic cells**	Days to weeks	DC turnover and precursor replenishment studies	Rapid turnover → low particulate accumulation, high sentinel activity
**Marginal zone macrophages**	Months	Studies on MZ macrophage renewal and stromal dependency	Slow renewal → stable filtering compartment for circulating particles
**Red pulp macrophages**	Months to years	Fate-mapping, parabiosis, macrophage self-renewal studies	Long-lived phagocytes → high capacity for particulate accumulation (microplastics, pigments, debris)
**Red pulp stromal cells**	Years	Stromal remodeling and ECM turnover studies	Very slow renewal → long-term retention of trapped particles

References

Splenic dendritic cells Not considered

Kamath, A. T., Henri, S., Battye, F., Tough, D. F., & Shortman, K. Developmental kinetics and lifespan of dendritic cells in mouse lymphoid organs. *Blood* **100(5)**, 1734–1741(2002). https://doi.org/10.1182/blood-2002-01-0042

Yona, S., Kim, K. W., Wolf, Y., Mildner, A., Varol, D., Breker, M., et al. Fate mapping reveals origins and dynamics of monocytes and tissue macrophages under homeostasis. *Immunity* **38(1**), 79–9 (2013). https://doi.org/10.1016/j.immuni.2012.12.001

Splenic macrophages (red pulp and marginal zone)

Hashimoto, D., Chow, A., Noizat, C., Teo, P., Beasley, M. B., Leboeuf, M., et al. Tissue-resident macrophages self-maintain locally throughout adult life with minimal contribution from circulating monocytes. *Immunity* **38(4)**, 792–804 (2013). https://doi.org/10.1016/j.immuni.2013.04.004

Stromal cells (red pulp / ECM)

Mebius, R. E., & Kraal, G. (2005). Structure and function of the spleen. Nature Reviews *Immunology* **5**, 606–616 (2013). https://doi.org/10.1038/nri1669

BONE MARROW — Cell Turnover, Sources, Bioenergetic Implications

Cell type (Bone Marrow)	Turnover	Typical source	Bioenergetic implication
Granulocyte–monocyte progenitors (GMPs)	Hours–days	Bone marrow proliferation/apoptosis studies	Extremely fast turnover → negligible accumulation; high ROS sensitivity
Multipotent progenitors (MPPs)	Days	Hematopoietic turnover quantification, apoptosis/PCNA labeling	Rapid cycling → high metabolic demand; low particulate retention
Megakaryocytes	5–10 days	Bone marrow niche studies, platelet production kinetics	Intermediate turnover → moderate exposure; potential interaction with microplastics before platelet release
Hematopoietic stem cells (HSCs)	Very slow; many remain quiescent for months	Studies on HSC quiescence, niche biology, single-cell mapping	Low turnover → minimal particulate accumulation; quiescence protects genome but increases vulnerability to chronic niche perturbation
Bone marrow stromal cells (MSC, CAR cells)	Months–years	Niche remodeling studies, inflammation-induced niche disruption	Very slow turnover → long-term retention of particles; key modulators of hematopoietic bioenergetics

References

Quantitative hematopoiesis overview

Cosgrove, J., Hustin, L. S. P., de Boer, R. J., & Perié, L. *Hematopoiesis in numbers*. Trends in *Immunology* **42(12),** 1100–1112 (2021) https://doi.org/10.1016/j.it.2021.10.005

Hematopoietic stem and progenitor cells (HSCs, MPPs, GMPs)

Swann, J. W., Zhang, R., Verovskaya, E. V., Calero-Nieto, F. J., Wang, X., Proven, M. A.,et al,. Inflammation perturbs hematopoiesis by remodeling specific compartments of the bone marrow niche. *Blood* **147(7)**, 739–754 (2026). https://doi.org/10.1182/blood.2025029513

Thiele, J., Zirbes, T. K., Lorenzen, J., Kvasnicka, H. M., Scholz, S., Erdmann, A.,et al. Hematopoietic turnover index in reactive and neoplastic bone marrow lesions: quantification by apoptosis and PCNA labeling. *Annals of Hematology* **75**, 33–39 (1997). https://doi.org/10.1007/s002770050262

Bernt, K. M. Mapping human hematopoiesis. *Nature Immunology*. (2024). https://doi.org/10.1038/s41590-024-01793-1

Bone marrow niche and stromal cells

Wang, Y., Deng, Z., Li, Y., Bai, K., Ma, J., Liu, Y., & Chen, Q. Function of hematopoiesis and bone marrow niche in inflammation and non-hematopoietic diseases. *Life Medicine* **4(3)**, lnaf015. (2025). https://doi.org/10.1093/lifemedi/lnaf015

PANCREAS — Cell Turnover, Sources, Bioenergetic Implications

Cell type (Pancreas)	Turnover	Typical source	Bioenergetic implication
β-cells (Islets)	Very slow; years; minimal replication in adults	Endocrine–exocrine crosstalk studies; β-cell lineage tracing	Extremely low turnover → high vulnerability to chronic metabolic stress; potential long-term particulate retention
α-cells (Islets)	Slow; months–years	Islet cell plasticity and lineage studies	Low turnover → moderate cumulative burden; stable glucagon output under stress
δ-cells / PP-cells	Slow	Endocrine cell mapping studies	Low turnover → stable paracrine signaling; low particulate clearance
Acinar cells (Exocrine)	Moderate; weeks	Exocrine regeneration and injury-repair studies	Higher turnover → lower particulate accumulation; high metabolic load due to enzyme synthesis
Ductal epithelial cells	Weeks–months	Pancreatic ductal lineage tracing	Intermediate turnover → moderate exposure; potential site for particulate trapping in ducts
Pancreatic stellate cells (PSC)	Very slow; years	Fibrosis and ECM remodeling studies	Extremely slow turnover → long-term retention of particles; major driver of fibrotic bioenergetic burden

References

Endocrine–exocrine crosstalk and turnover *(years)*

Overton, D. L., and Mastracci, T. L. Exocrine–endocrine crosstalk: The influence of pancreatic cellular communications on organ growth, function and disease. Frontiers in Endocrinology **13**, 904004 (2022). https://doi.org/10.3389/fendo.2022.904004

Hu, C., Chen, Y., Yin, X., Xu, R., Yin, C., Wang, C., Zhao, Y. Pancreatic endocrine and exocrine signaling and crosstalk in physiological and pathological status. *Signal Transduction and Targeted Therapy*. (2023). https://doi.org/10.1038/s41392-024-02098-3

Exocrine–endocrine interactions and structural turnover *weeks-months*

Valente, R., Coppola, A., Scandavini, C. M., Halimi, A., Magnusson, A., Lauro, A., Sotirova, I., Arnelo, U., Franklin, O. Interactions between the exocrine and the endocrine pancreas. *Journal of Clinical Medicine* **13(4)**, 1179 (2024). https://doi.org/10.3390/jcm13041179

Mostafa, A., Gantsova, E. A., Serova, O. V., Mohammad, T., Deyev, I. E.). Interaction between endocrine and exocrine pancreas**.** *Journal of Evolutionary Biochemistry and Physiology* **60**, 792–801 (2024). httpss//doi,org/10.1134/S0022093024020273

VASCULAR ENDOTHELIUM — Cell Turnover, Sources, Bioenergetic Implication

Cell type / Organ	Turnover	Typical source	Bioenergetic implication
Vascular endothelium (systemic microvasculature)	Weeks to months (variable by vascular bed; accelerated under inflammation, shear stress, or metabolic disease)	Endothelial progenitors; local endothelial self-renewal; shear-dependent remodeling	High mitochondrial and glycolytic demand for barrier integrity, nitric oxide production, and redox signaling. Continuous exposure to circulating xenobiotics → rapid oxidative exhaustion. Microplastics induce endothelial activation, mitochondrial dysfunction, and impaired NO bioavailability → early vascular aging.
Arterial endothelium (large vessels)	Slow (months; turnover increases with disturbed flow)	Hemodynamic stress–driven renewal; local endothelial plasticity	High ATP requirement for mechanotransduction and anti-inflammatory signaling. Disturbed flow amplifies ROS generation → microplastics accelerate endothelial senescence and atherogenic remodeling.
Capillary endothelium	Moderate (weeks)	Tissue-specific microvascular niches; pericyte–endothelial crosstalk	Metabolically flexible cells with tight redox control. Persistent microplastic exposure disrupts mitochondrial dynamics and increases permeability → chronic tissue-level oxidative burden.
Venous endothelium	Moderate–slow	Low-shear vascular beds; inflammatory recruitment	Lower shear stress → reduced antioxidant defenses. Microplastics accumulate longer in low-flow regions → sustained endothelial activation and impaired barrier recovery.

References

Microvasculature -*weeks-months*

Krüger-Genge, A., Blocki, A., Franke, R.-P., & Jung, F. Vascular endothelial cell biology: An update. *International Journal of Molecular Sciences* **20(18)**, 4411 (2019). https://doi.org/10.3390/ijms20184411

Capillary endothelium (microvascular niches) *weeks*

Fonseca, C. G., Barbacena, P., & Franco, C. A. Endothelial cells on the move: Dynamics in vascular morphogenesis and disease. *Vascular Biology* **2(1)**, H29–H43 (2020). https://doi.org/10.1530/VB-20-0007

Arterial endothelium (large vessels) *months*

Li, Y., Liu, Z., Han, X., Liang, F., Zhang, Q., Huang, X., et al. Dynamics of endothelial cell generation and turnover in arteries during homeostasis and diseases. *Circulation* (2021). https://doi.org/10.1161/CIRCULATIONAHA.120.050234

Venous endothelium *weeks-months*

Grego, A., Fernandes, C., Fonseca, I., Dias-Neto, M., Costa, R., Leite-Moreira, A., et al. Endothelial dysfunction in cardiovascular diseases: Mechanisms and in vitro models. *Molecular and Cellular Biochemistry* **480**, 4671–4695 (2025). https://doi.org/10.1007/s11010-025-04936-3

TISUE RESIDENT MACROPHAGES— Turnover, Sources, Bioenergetic Implication

Macrophage population	Turnover	Typical source	Bioenergetic implication
Kupffer cells (Liver)	Months–years; self-renewing	Fate-mapping, parabiosis, embryonic origin studies	Long-lived phagocytes → major reservoir for persistent particles (microplastics, pigments, metals)
Red pulp macrophages (Spleen)	Months–years	Tissue-resident macrophage renewal studies	Slow turnover → high particulate retention; central role in RBC clearance and iron recycling
Marginal zone macrophages (Spleen)	Months	Stromal-dependent renewal studies	Stable filtering compartment → sustained exposure to circulating particles
Alveolar macrophages (Lung)	Years; extremely slow	Embryonic origin + self-renewal studies	Very long-lived → cumulative particulate burden (PM, microplastics, carbon)
Microglia (Brain)	Years–decades	^14C birth-dating, lineage tracing	Ultra-slow turnover → long-term retention; high vulnerability to chronic inflammatory or particulate stress
Langerhans cells (Skin)	Months–years	Epidermal renewal and LC self-maintenance studies	Slow turnover → persistent antigen/particle retention in epidermal niche
Intestinal macrophages	Days–weeks	Monocyte-derived replenishment studies	Rapid turnover → low particulate accumulation; high metabolic flux due to constant microbial exposure

References

Core tissue-resident macrophage biology

Hashimoto, D., Chow, A., Noizat, C., Teo, P., Beasley, M. B., Leboeuf, M., et al. Tissue-resident macrophages self-maintain locally throughout adult life with minimal contribution from circulating monocytes. *Immunity* **38(4),** 792–804 (2013). https://doi.org/10.1016/j.immuni.2013.04.004

Yona, S., Kim, K. W., Wolf, Y., Mildner, A., Varol, D., Breker, M.et al. Fate mapping reveals origins and dynamics of monocytes and tissue macrophages under homeostasis. *Immunity* **38(1)**, 79–91 (2013). https://doi.org/10.1016/j.immuni.2012.12.001

Lung (alveolar macrophages)

Guilliams, M., De Kleer, I., Henri, S., Post, S., Vanhoutte, L., De Prijck, S., et al. Alveolar macrophages develop from fetal monocytes that differentiate into long-lived cells in the first week of life via GM-CSF. *Journal of Experimental Medicine* **210(10)**, 1977–1992 (2013). https://doi.org/10.1084/jem.20131199

Brain (microglia)

Reu, P., Khosravi, A., Bernard, S., Mold, J. E., Salehpour, M., Alkass, K.et al.). The lifespan and turnover of microglia in the human brain. *Cell Reports* **20(4)**, 779–784 (2017). https://doi.org/10.1016/j.celrep.2017.07.004

Skin (Langerhans cells)

Merad, M., Ginhoux, F., & Collin, M. Origin, homeostasis and function of Langerhans cells and other Langerin-expressing dendritic cells. *Nature Reviews Immunology* **8**, 935–947 (2008). https://doi.org/10.1038/nri2455

Intestinal macrophages

Bain, C. C., & Mowat, A. M.). Macrophages in intestinal homeostasis and inflammation. *Immunological Reviews* 260(1), 102–117 (2014). https://doi.org/10.1111/imr.12192

TESTIS— Cell Turnover, Sources, Bioenergetic Implications

Cell type (Testis)	Turnover	Typical source	Bioenergetic implication
Spermatogonia (SSC + differentiating)	Days–weeks	Spermatogenesis kinetics; SSC renewal studies	Rapid turnover → high metabolic flux; minimal particulate accumulation
Primary spermatocytes	~23 days (human)	Meiotic progression studies	Intermediate turnover → moderate metabolic load during meiosis
Spermatids → Spermatozoa	~21–23 days	Spermiogenesis timing studies	Rapid differentiation → low particulate retention; high mitochondrial remodeling
Sertoli cells	Very slow; years	Sertoli cell stability and lineage studies	Extremely low turnover → long-term retention of particles; high bioenergetic burden due to constant support of germ cells
Leydig cells	Months	Steroidogenic cell renewal studies	Slow turnover → cumulative oxidative burden from chronic testosterone synthesis
Peritubular myoid cells	Months–years	Testicular niche remodeling studies	Very slow turnover → potential long-term particulate retention; structural role in seminiferous tubule integrity
Testicular macrophages	Months–years	Tissue-resident macrophage studies	Long-lived → reservoir for persistent particles; key modulators of steroidogenesis

References

Spermatogenesis and germ cell turnover -*weeks*

Tirumalasetty, M. M. B., Bhattacharya, I., Mohiuddin, M. S., Baki, V. B., & Choubey, M. Understanding testicular single cell transcriptional atlas: from developmental complications to male infertility. *Frontiers in Endocrinology* **15,** 1394812. (2024). https://doi.org/10.3389/fendo.2024.1394812

Sertoli -*years*

Guo, J., Nie, X., et al.. The dynamic transcriptional cell atlas of testis development during human puberty. *Cell Stem Cell* **26(2)**, 262–276.e4 (2020). https://doi.org/10.1016/j.stem.2019.12.005

Muscle – Turnover, Sources, Bioenergetic Implications

Cell type	Turnover	Typical Source	Bioenergetic Implication
**Myofibers (skeletal muscle fibers)**	Slow cellular turnover (myonuclei are long-lived; protein turnover is continuous but not rapid)	Satellite cells, myonuclear domain maintenance, contractile protein renewal	High ATP demand for protein synthesis, mitochondrial maintenance, and excitation–contraction coupling; vulnerability increases when oxidative phosphorylation is impaired
**Skeletal muscle myocytes**	Protein half-life varies (myofibrillar proteins ~5–30 days; mitochondrial proteins shorter)	Continuous proteostasis via ubiquitin–proteasome system and autophagy–lysosome pathways	High bioenergetic cost for proteostasis; mitochondrial dysfunction rapidly affects force generation and recovery
**Myofibers + Satellite**	Satellite cell activation is episodic (injury, hypertrophy)	Pax7+ satellite cells	Regeneration requires intense metabolic activation, switching from quiescence (low ATP turnover) to proliferation (high glycolytic and oxidative demand)

References

Blau, H. M., Cosgrove, B. D., & Ho, A. T. V. The central role of muscle stem cells in regenerative failure with aging. *Nature Medicine* **21**, 854–862 (2015), https://doi.org/10.1038/nm.3918

Hwee, D. T., et al. Mitochondrial protein turnover in skeletal muscle. *Journal of Applied Physiology* **116(7**), 880–888 (2014). https://doi.org/10.1152/japplphysiol.01395.2013

details for the final framework evaluation

The Ma content, BMRt, and ISVI sensitivity of the 19 organ/tissue are summarized in Table S6

Table S6. Single variables score and total score of framework evaluation

Organ / Tissue	Ma	BMRt	ISVI	Total
Bone marrow	4	5	3	12
Spleen	5	3	3	11
Lung (alveolar epithelium)	4	3	4	11
Intestinal epithelium	3	5	3	11
Liver	4	3	3	10
Kidney	3	3	4	10
Pancreas	3	3	4	10
Vascular endothelium	3	4	3	10
Placenta	**3**	4	3	10
Testis	2	3	5	10
Skin (epidermis)	3	4	2	9
Heart	2	2	5	9
Uterus	3	3	3	9
Ovaries	2	2	4	8
Thyroid	2	3	3	8
Brain	1	1	5	7
Adipose tissue	3	2	2	7

12 Points

Bone marrow ranks highest scoring 12 and share a unique constellation of bioenergetic and structural features that make it the most vulnerable and most metabolically demanding in the entire organism.

11 Points

Each organ is a **hub**:

- Liver: metabolic hub
- Lung: respiratory hub
- Bone marrow: hematopoietic hub
- Spleen: immunological hub
- Neurons: integrative hub

Implication: Their failure produces immediate systemic consequences.

High perfusion dependency

All five organs rely on **continuous, high-flow perfusion**:

- Liver: dual blood supply
- Lung: pulmonary circulation
- Spleen: slow but constant filtration
- Bone marrow: sinusoidal perfusion

- Brain: autoregulated, high-demand circulation

Implication: Microvascular dysfunction (e.g., microplastics, inflammation) disproportionately affects them.

The 11-point organs form a cluster characterized by:

High metabolic throughput, high oxygen exposure, high turnover, systemic centrality, and perfusion dependency.

10 points

The intestine, kidney, and skeletal muscle all have high flux, ATP-dependent transport physiology, indicating that they constantly transport huge quantities of water, ions, nutrients, and metabolites across large epithelial or membrane surfaces. To maintain homeostasis, they depend on tight perfusion, rapid solute cycling, and sustained mitochondrial activity.

9 points

What the 9-point organs have in common

With continuous metabolic activity, hormone or barrier-related functions, and prolonged exposure to oxidative or inflammatory stress, they all have a moderately high but not extreme bioenergetic demand. Nevertheless, they also exhibit adequate structural turnover or functional redundancy to prevent the critical vulnerability reported in higher ranked organs.

>10 <

Bioenergetic Threshold Interpretation (>10 vs ≤10)

Organs with scores higher than 10 are classified as high priority bioenergetic systems, which are characterised by limited tolerance to decline in function, rigorous dependence on oxygen delivery, and continuous metabolic throughput. Even minor alterations in mitochondrial efficiency, perfusion, or redox balance may have significant systemic effects due to these tissues function closely to the maximum limits of physiological energy flux.

Organs possessing a score of 10 or below, on the other hand, have intermediate bioenergetic demands, which are amplified by increased structural turnover, partial functional redundancy, or more flexible metabolic strategies. These tissues have a greater ability for adaptation and do not represent the same immediate systemic risk when challenged, even if they are still susceptible to energetic stress.

S7 Bioenergetic Damage Panel (3-marker framework)

A minimal set of four blood-based variables can capture early bioenergetic distress across the organs ranked in the upper tiers of the matrix (>10), integrating oxidative load, perfusion mismatch, cytolytic stress, and myocardial vulnerability.

1. Plasma lactate

A sensitive indicator of impaired oxidative phosphorylation and oxygen-delivery mismatch, capturing early failure in high-throughput, oxygen-dependent tissues such as myocardium, neurons, liver, and alveolar epithelium.

Lactate as a marker of oxygen-delivery mismatch and metabolic stress

Bakker, J., & Jansen, T. C. Don't take vitals, take a lactate. *Intensive Care Medicine* **33(11)**, 1863–1865 (2007). https://doi.org/10.1007/s00134-007-0680-6

Lactate as a systemic indicator of impaired oxidative phosphorylation

Kraut, J. A., & Madias, N. E. Lactic acidosis. *New England Journal of Medicine* **371(24)**, 2309–2319 (2014). https://doi.org/10.1056/NEJMra1309483

Consistent with the role of plasma lactate as an early indicator of oxygen-delivery mismatch (Bakker & Jansen, 2007) and impaired oxidative phosphorylation under metabolic stress (Kraut & Madias, 2014), elevated lactate provides a sensitive, system-level readout of bioenergetic strain across high-throughput organs scoring ≥ 10 in the matrix.

2. Serum LDH

A broad cytolysis marker represents structural stress in the central range of the matrix and integrates damage across metabolically active or high turnover organs (liver, lung, spleen, bone marrow, gut, skeletal muscle).

In high throughput, high turnover tissues, cytosolic stress can be observed in how LDH fits into the bioenergetic framework and why it has nothing to do with cholesterol. LDH identifies a common vulnerability across organs scoring ≥10: these organs function with strong glycolytic–mitochondrial connection, fast metabolite flow, and ongoing structural renewal. LDH is a sensitive integrator of bioenergetic strain because even little changes in perfusion, redox balance, or cellular integrity cause minimal but observable leakage.

LDH reflects cytosolic stress in high-flux, metabolically active organs—not lipid overload—making it a coherent marker of bioenergetic strain across all tissues scoring ≥10.

LDH as a regulator of the lactate–pyruvate shuttle and redox balance

Brooks, G. A. The science and translation of lactate shuttle theory. *Cell Metabolism* **27(4),** 757–785 (2018). https://doi.org/10.1016/j.cmet.2018.03.008

LDH as a marker of metabolic stress and glycolytic compensation

San-Millán, I., and Brooks, G. A. (2017). Reexamining cancer metabolism: Lactate production for carcinogenesis could be the purpose of the Warburg effect. *Carcinogenesis* **38(2)**, 119–133. https://doi.org/10.1093/carcin/bgw127

3. High-sensitivity CRP (hs-CRP)

A sensitive indicator of systemic inflammatory activation that increases oxidative and metabolic stress in the ≥10 group and disproportionately affects high throughput, perfusion-dependent organs. The inflammatory component of the MIC Syn bioenergetic triad is represented by hsCRP. Contrary to lactate and LDH, which indicate cytolytic turnover and metabolic overload, hsCRP reveals the systemic immune activation brought on by stress resulting from MNPs. The organism has transitioned from a localised metabolic disruption to a coordinated inflammatory response, demonstrated by its increase. Due to these factors, hsCRP is crucial to MIC Syn's diagnostic interpretation.

When hsCRP, lactate, and LDH all exceed their standardised pathological limits, indicating concurrent metabolic, cytolytic, and inflammatory activation, the syndrome is subsequently considered to be existent.

As opposed to functioning as inert particles, MPs containing conductive additives behave as intracellular electroactive entities. After internalisation, they dissipate ionic gradients, distort local electric fields, and function as charge distribution sinks. In order to maintain calcium homeostasis, pump ions, and restore membrane potentials, the cell must constantly use ATP, which results in pointless kinetic energy cycling.

Simultaneously, by altering surface charge, encouraging lipid peroxidation, and raising local permeability, the conductive surface of the particles disrupts the stability of intracellular membranes, especially lysosomal and mitochondrial membranes. Reduced $\Delta\Psi m$, decreased oxidative phosphorylation, and increased ROS generation result from the vulnerability of the inner membrane of the mitochondria.

In summary conductive MPs combine structural instability of critical bioenergetic membranes with useless ATP consumption to generate a novel kind of electro-bioenergetic stress. This dual mechanism increases cellular susceptibility to oxidative and inflammatory damage while decreasing metabolic efficiency.
